# Magnitude and heterogeneity of brain structural abnormalities in 22q11.2 deletion syndrome: a meta-analysis

**DOI:** 10.1038/s41380-019-0638-3

**Published:** 2020-01-10

**Authors:** Maria Rogdaki, Maria Gudbrandsen, Robert A McCutcheon, Charlotte E Blackmore, Stefan Brugger, Christine Ecker, Michael C Craig, Eileen Daly, Declan G M Murphy, Oliver Howes

**Affiliations:** 1 Department of Psychosis Studies, Institute of Psychiatry, Psychology and Neuroscience, King’s College London, London, SE5 8AF UK; 2 Psychiatric Imaging Group, MRC London Institute of Medical Sciences, Imperial College, London, W12 0NN UK; 3 Department of Forensic and Neurodevelopmental Sciences, and the Sackler Institute for Translational Neurodevelopmental Sciences, Institute of Psychiatry, Psychology and Neuroscience, King’s College, London, SE5 8AF UK; 4 Cardiff University Brain Research Imaging Centre, School of Psychology, Cardiff University, Cardiff, Wales CF24 4HQ UK; 5 Division of Psychiatry, UCL, Maple House, London, W1T 7NF UK; 6 Department of Child and Adolescent Psychiatry, Psychosomatics and Psychotherapy, University Hospital Frankfurt am Main, Goethe-University Frankfurt am Main, Frankfurt, Germany; 7 National Autism Unit, Bethlem Royal Hospital, London, UK; 8 MRC Centre for Neurodevelopmental Disorders, King’s College London, London, UK

**Keywords:** Neuroscience, Psychiatric disorders

## Abstract

The 22q11.2 deletion syndrome (22q11.2DS) is a neurodevelopmental disorder associated with a number of volumetric brain abnormalities. The syndrome is also associated with an increased risk for neuropsychiatric disorders including schizophrenia and autism spectrum disorder. An earlier meta-analysis showed reduced grey and white matter volumes in individuals with 22q11.2DS. Since this analysis was conducted, the number of studies has increased markedly, permitting more precise estimates of effects and more regions to be examined. Although 22q11.2DS is clinically heterogeneous, it is not known to what extent this heterogeneity is mirrored in neuroanatomy. The aim of this study was thus to investigate differences in mean brain volume and structural variability within regions, between 22q11.2DS and typically developing controls. We examined studies that reported measures of brain volume using MRI in PubMed, Web of Science, Scopus and PsycINFO from inception to 1 May 2019. Data were extracted from studies in order to calculate effect sizes representing case–control difference in mean volume, and in the variability of volume (as measured using the log variability ratio (lnVR) and coefficient of variation ratio (CVR)). We found significant overall decreases in mean volume in 22q11.2DS compared with control for: total brain (*g* = −0.96; *p* < 0.001); total grey matter (*g* = −0.81, *p* < 0.001); and total white matter (*g* = −0.81; *p* < 0.001). There was also a significant overall reduction of mean volume in 22q11.2DS subjects compared with controls in frontal lobe (*g* = −0.47; *p* < 0.001), temporal lobe (*g* = −0.84; *p* < 0.001), parietal lobe (*g* = −0.73; *p* = 0.053), cerebellum (*g* = −1.25; *p* < 0.001) and hippocampus (*g* = −0.90; *p* < 0.001). Significantly increased variability in 22q11.2DS individuals compared with controls was found only for the hippocampus (VR, 1.14; *p* = 0.036; CVR, 1.30; *p* < 0.001), and lateral ventricles (VR, 1.56; *p* = 0.004*)*. The results support the notion that structural abnormalities in 22q11.2DS and schizophrenia are convergent, and also to some degree with findings in autism spectrum disorder. Finally, the increased variability seen in the hippocampus in 22q11.2DS may underlie some of the heterogeneity observed in the neuropsychiatric phenotype.

## Introduction

22q11.2 deletion syndrome (22q11.2DS), also known as DiGeorge or Velo-Cardio-Facial syndrome, is a neurogenetic disorder with an estimated prevalence ranging between 1:3000 and 1:6000 [1] and is the most commonly occurring microdeletion in humans [1–3]. About 90% of the cases arise from de novo mutations, whilst about 10% are inherited in an autosomal dominant pattern. The 22q11.2 locus is one of the most complex regions in the genome due to the large clusters of low copy repeats (LCR), which predict genomic instability [4, 5]. Non allelic homologous recombination between LCRA and LCRD, the two largest LCRs, leads to a 3 Mb deletion and accounts for 90% cases, whereas recombination of LCRA and LCRB or LCRA and LCRC leads to 1.5 and 2 Mb deletions, respectively [4–6]. It has been suggested that variability in the deletion size and breakpoint locations, as well as characteristics of the intact chromosome are likely to be playing an important role in the clinical phenotype of individuals with 22q11.2DS [7]. The physical manifestations have their onset in early childhood and include abnormalities of the cardiac, endocrine and immune systems in addition to facial deformities and cleft palate [1, 8]. Furthermore, anxiety disorders are prevalent across the lifespan and by adolescence mood disorders emerge [9]. In addition, neurodevelopmental disorders such as autism spectrum disorder (ASD) have estimated prevalence rates between 18 and 58% [9–12]. Over the last 20 years, it has been well established that individuals with 22q11.2 deletion have greater than 25% risk of developing psychosis [9], making this copy number variant one of the strongest risk factors for the development of psychosis [13, 14].

Given the neuropsychiatric sequelae of the syndrome, there has been considerable interest in brain structure in 22q11.2DS. There have been two meta-analyses on structural neuroimaging studies in 22q11.2DS to date. The first meta-analysis reported global volumetric reductions of total grey and white matter, as well as reductions in frontal cortex and hippocampal volume [15]. Authors further found that the magnitude of the effect sizes increased from frontal towards the occipital regions [15], supporting the theory that brain structural abnormalities in 22q11.2DS may reflect neurodevelopmental pathology along the rostrocaudate gradient [16]. More recently Sun et al. conduced a large multicentre study, examining cortical grey matter, and showed overall decreased volume, driven by reduced surface area, and increased cortical thickness, with the exception of the temporal pole [17].

As 22q11.2DS confers significant risk for both schizophrenia and ASD, the question as to whether they share similar structural abnormalities has been repeatedly asked [10, 12, 18–25]. Moreover, in 22q11.2DS, the physical and psychiatric consequences of the deletion are highly variable, both in terms of nature and degree of symptomatology, suggesting that multiple neurobiological pathways may mediate the relationship between genes and phenotypic expression [7]. Therefore, when examining the brain structure, it is also important to consider the *variability* of brain regions volumes. This enables the examination of whether the clinical heterogeneity of the disorder is similarly reflected at the level of neuroanatomy and whether the evidence of differences in subgroups is a false positive finding deriving from the selection of individuals from extreme ends of distribution of similar variance to that of healthy volunteers, but with shifted mean. Variability is a relatively new concept, which was examined recently by a meta-analysis in idiopathic schizophrenia [26]. Although one might expect similar neuroanatomical variability to be present in 22q11.2DS, this has not yet been tested.

Since the publication of an initial meta-analysis of structural brain differences by Tan et al. [15], including studies up to March 2008, the total number of structural magnetic resonance studies in 22q11.2DS has almost doubled, and to our knowledge, no previous study has examined variability of brain region volumes in 22q11.2DS. This study therefore aimed to (1) perform an updated meta-analysis of mean volume differences between individuals with 22q11.2DS and typically developing controls; and (2) examine differences in brain structural variability between groups. We hypothesised that there would be volumetric reductions in total white and grey matter, as well as in cortical and subcortical regions including frontal, temporal, parietal lobe and hippocampus, and further that neuroanatomical variability would be greater in 22q11.2DS compared with controls.

## Materials and methods

### Study selection

A comprehensive search was conducted of PubMed, Web of Science, Scopus and PsycINFO from inception to 1st May 2019. Search terms used to identify the studies included: 22q11.2 DS OR DiGeorge OR Velo-Cardio-Facial syndrome OR Shprintzen syndrome OR CATCH22, Conotruncal anomaly face syndrome; AND magnetic resonance (MRI) OR volume OR SBM OR seed OR morphology OR morphometry OR gray/grey OR cortical OR anatomy OR structur* OR brain. In addition, we supplemented the search by manual and bibliographic cross referencing, and by examining the previous meta-analysis [15] to identify potentially missed studies (see Supplementary Fig. 1 for flow chart).

Studies were initially included if they were (1) published as a peer-reviewed article with original data and reported measures of regional brain volumes in individuals with 22q11.2DS and typically developing controls; (2) had sufficient data to extract mean and standard deviations for both groups; and (3) were written in English. For papers with missing information, authors were contacted for raw data and/or means and standard deviations.

In papers where samples overlapped, we only included the study with the largest participant size. However, some of the smaller studies included regions that were not covered in the larger paper and, if so, these duplicate samples remained included for the missing regions, but weighted by the smaller participant number. The search, screening and data extraction was completed independently by three separate researchers, MR, MG and CEB.

Measures reported by subgroups (e.g. male vs. female or two control groups) were included as separate results. To avoid overweighting, in cases where a control group was used multiple times against different patient groups, the number of participants recorded for that studies’ control group was reduced accordingly, in line with standard guidelines [27]. Where studies presented left and right hemisphere volumes separately, these were combined to a single measure, as previously described [28], using correlation coefficients derived from an existing dataset (see Supplementary Table 1).

Means and standard deviations of volumetric measures for both patient and control groups were extracted. Brain structures were included in the analysis if at least three studies met the inclusion criteria. Further, we recorded details of the potential moderating factors, such as sex and IQ.

### Outcome measures for mean differences

A meta-analysis of between-group differences in mean volumes was conducted, indexed using Hedges *g*.

### Outcome measures for variability

We measured the relative variability of brain regions in patients compared with controls, by using the log variability ratio (InVR): [26, 29]

$$\ln {\it{VR}}\,=\,\ln \left( {\frac{{\sigma _{\rm{p}}}}{{\sigma _{\rm{c}}}}} \right)\,=\,ln\left( {\frac{{S_{\rm{p}}}}{{S_{\rm{c}}}}} \right)\,+\,\frac{1}{{2(n_{\rm{p}}\,-\,1)}}\,-\,\frac{1}{{2(n_{\rm{c}}\,-\,1)}},$$

where *σ*_p_ and *σ*_c_ are unbiased estimates of population standard deviations, *S*_p_ and *S*_c_ are reported samples standard deviations, and *n*_p_ and *n*_c_ are the sample sizes for patient and control. However, as variance is positively correlated with mean, some of the between-group difference in relative variability might be partly driven by between-group differences in the mean. This is in particular true for brain structures with larger mean volume in patients, such as lateral ventricles. We therefore also calculated the log coefficient of variation ratio (lnCVR), which is a more conservative test [26, 29]. The latter term, measures variability differences after accounting for differences in mean:

$$\ln {\it{CVR}}\,=\,\ln \left( {\frac{{\sigma _{\rm{p}}/\bar x_{\rm{p}}}}{{\sigma _{\rm{c}}/\bar x_{\rm{c}}}}} \right)\,=\,\ln\left( {\frac{{S_{\rm{p}}/\bar x_{\rm{p}}}}{{S_{\rm{c}}/\bar x_{\rm{c}}}}} \right)\,+\,\frac{1}{{2(n_{\rm{p}}\,-\,1)}}\,-\,\frac{1}{{2(n_{\rm{c}}\,-\,1)}},$$

where $\bar x_{\mathrm{p}}$ and $\bar x_{\mathrm{c}}$ are the reported means for patients and controls.

### Statistical analysis

As many of our studies reported volumes for multiple structures of interest, we used a multivariate approach, enabling an estimation of summary effect sizes across all regions of interest, while reducing multicity concerns [30]. This approach estimates covariance among outcome measures, thereby improving estimation of summary effect size relative to univariate analysis [31].

All analyses were conducted using the metafor package in R (3.3.2) [32]. Separate multivariate random effect models for Hedges’ g, lnVR and lnCVR were performed so as to conduct meta-analysis of all regions concurrently [32]. We added random effects to the model for each region within each study and region was added to the model as a categorical moderator to derive summary effect size for regions separately. Random effects models were then fitted to data in all regions by means of restricted maximum likelihood estimation for Hedges g, lnVR and lnCVR. A Wald-type chi-square omnibus test was performed to assess the significance of the model coefficients across regions.

For each significant omnibus test, we tested the effect separately by region. Where VR (or CVR) was 1, equal variability between patients and controls were found, whereas >1 indicated greater variability in the patient group, and <1 indicated lower variability in the patient group.

In addition, an omnibus test of effect of moderators was used to examine the overall effect of region on mean values and subsequently post hoc tests were applied to assess differences between regions at pairwise level. In view of the number of tests we conducted, we employed false discovery rate adjustment of the probability threshold to the expected proportion of type I error to 5% of rejected null hypotheses.

### Meta-regression/sensitivity analysis

To examine the effects of moderating factors on mean differences and variability, we employed a univariate mixed effects meta-regression. We separately examined age, sex and IQ as moderators for the mean volume differences and variability. No correction for multiple comparisons was applied when assessing effects at the level of the individual region, as these meta-regression analyses were exploratory.

Eleven studies in the meta-analysis included individuals with 22q11.2DS with psychiatric comorbidities (e.g. psychosis, ASD, ADHD, anxiety and mood disorder) or on psychotropic medication. To test whether diagnosis had an influence on our results, we repeated the analysis for mean volume differences and variability only for individuals with 22q11.2DS without psychiatric comorbidities.

### Publication bias and inconsistency for meta-analysis of mean differences

Publication bias was assessed across all regions simultaneously by inspection of funnel plots of standard errors against regional residuals and by a multivariate analogue of Egger’s regression test [33]. Inconsistency between studies was evaluated using the *I*^2^ statistic (with >50% conventionally indicating moderate–high inconsistency and <50% indicating low–moderate inconsistency [34]), an approach that generalises straightforwardly to the multivariate setting [35].

## Results

### Study selection

A total of 24 studies, reporting data from 988 individuals with 22q11.2DS and 873 controls were included (see Table 1). Inter-rater reliability was assessed using Fleiss Kappa test. The kappa was 0.9 for included studies and 0.94 for data extraction, reflecting excellent between rater reliability. Sufficient studies were found to conduct analyses for the following regions: total brain, total grey matter, total white matter, cerebral spinal fluid, frontal, temporal and parietal lobes, cerebellum, lateral ventricles, caudate nucleus, amygdala and hippocampus. Mean (standard deviation) age for individuals with 22q11.2DS was 14.4 (5.8) years and for controls 14.5 (6.6), and the male:female percentage (%) was 50:50 in the 22q11.2DS group and 53:47 in the control group.

**Table 1:** Studies included in the meta-analysis.

Study	Genetic information	Diagnostic information	Type of scanner	Method	Brain region	Participants (*n*)		IQ				Age				Gender			
						22q11DS	Controls	22q11DS		Controls		22q11DS		Controls		22q11DS		Controls	
								Mean	SD	Mean	SD	Mean	SD	Mean	SD	Male	Female	Male	Female
Antshel et al. 2005 [71]	FISH^a,b^	ADHD in all groups: 22q11.2DS—18 males and 10 females; controls —4 males and 1 female; siblings—0 males and 1 female	1.5T	Brain Image	Frontal lobe, parietal lobe, cerebellum,	25	28	68.9	12.8	95.6	10.3	11.1	2.7	10.7	2.4	25	n/a	28	n/a
						25	13	68.9	12.8	103.0	15.0	11.1	2.7	12.3	1.9	25	n/a	13	n/a
						20	19	76.3	11.7	97.5	13.6	10.8	2.5	9.2	2.3	n/a	20	n/a	19
						20	17	76.3	11.7	105.9	13.1	10.8	2.5	12.2	1.9	n/a	20	n/a	17
Antshel et al. 2008 [72]	FISH^a,b^	No diagnostic information available	1.5T	Brain Image	Total brain	92	59	71.7	12.9	97.1	12.6	11.2	2.6	10.4	2.6	51	41	34	25
Baker et al. 2011 [73]	FISH^a,b^	No major psychiatric disorder	1.5T	SPM/MRIcron	Total grey matter, total white matter, hippocampus, CFS, amygdala, caudate	7	13	n/a	n/a	n/a	n/a	17.7	2.1	19.3	2.3	3	4	6	7
						7	14	n/a	n/a	n/a	n/a	17.7	2.1	17.8	1.9	4	3	7	7
Bearden et al. 2004 [74]	FISH^a,b^	22q11.2DS group: 1 psychotic disorder and pervasive developmental disorder	1.5T	Segmentation handtracing	Frontal lobe, temporal lobe	13	9	80.8	16.1	118.3	15.0	12.3	3.2	12.2	2.8	6	7	5	4
Bearden et al. 2007 [75]	FISH^a,b^	22q11.2DS group: 6 ADHD; 1 oppositional defiant disorder; 1 PDD/psychosis-NOS; 10 anxiety disorders; 1 anxiety disorder-NOS; 4 specific phobia; 1 social phobia/GAD; 1 OCD traits; 1 PTSD/ acute stress disorder and 1 panic disorder	1.5T	LONI pipeline	Total brain	21	13	74.5	14.6	111.3	12.3	11.7	2.8	10.9	2.6	10	11	7	6
Bearden et al. 2009 [76]	FISH^a,b^	No diagnostic information available	1.5T	LONI pipeline	Total brain	21	13	74.5	14.6	111.3	12.3	11.7	2.8	10.9	2.6	10	11	7	6
Campbell et al. 2006 [77]	FISH^a,b^	SDQ and ASQ scales used but no information about diagnosis provided	1.5T	SPM	Total brain, frontal lobe, temporal lobe, parietal lobe, caudate	39	26	67.0	10.0	102.0	12.0	11.0	3.0	11.0	3.0	20	19	16	10
Chow et al. 2002 [78]	FISH^a,b^	22q11.2DS group: 11 schizophrenia and 3 schizoaffective disorder	1.5T	Brain Image	Total grey matter, total white matter, CFS, lateral ventricles	13	13	71.3	10.0	116.2	6.7	27.5	6.4	28.2	6.6	7	6	7	6
Debbane et al. 2006 [79]	FISH^a,b^	No major psychiatric disorder	1.5T	Brain Image	Total grey matter, total white matter, total brain, hippocampus, amygdala	43	40	69.4	11.5	111.1	13.4	16.7	8.7	15.1	7.9	16	27	17	23
Deboer et al. 2007 [65]	FISH^a,b^	No diagnostic information available	1.5T	SPM	Total grey matter, total white matter, CFS	36	36	77.0	12.0	110.0	11.0	10.8	2.3	10.5	1.9	17	19	23	13
Dufour et al. 2008 [68]	PCR sequencing	22q11.2DS group: 24 psychosis (5 schizophrenia and 10 on psychotropic medication) and 18 non psychotic	1.5T	Brain Image	Total brain	58	64	69.0	11.8	111.9	13.0	15.5	8.8	15.0	8.1	25	33	25	39
Eliez et al. 2000 [66]	FISH^a,b^	No diagnostic information available	1.5T	Brain Image	Frontal lobe, parietal lobe, cerebellum,	15	15	n/a	n/a	n/a	n/a	10.5	3.1	10.8	2.7	10	5	10	5
Eliez et al. 2001 [80]	FISH^a,b^	No diagnostic information available	1.5T	Brain Image	Total brain, temporal lobe, hippocampus, amygdala	23	23	n/a	n/a	n/a	n/a	12.7	3.9	12.9	4.1	15	8	15	8
Eliez et al. 2002 [81]	FISH^a,b^	22q11.2DS group: 1 mood disorder and 1 psychosis (neuroleptic medication)	1.5T	Brain Image	Total grey matter, total white matter, caudate	30	30	69.5	16.7	115.9	11.6	12.1	3.8	12.2	4.4	n/a	n/a	n/a	n/a
Glaser et al. 2007 [82]	FISH^a,b^	22q11.2DS group: 21 Hallucinations or delusions and 1 schizophrenia (treated at time of the scanning)	1.5T	BRAINS	Total brain	42	54	n/a	n/a	n/a	n/a	14.0	5.1	13.4	5.5	18	24	23	31
Gothelf et al. 2007 [83]	FISH^a,b^	22q11.2DS group: T1—No major psychiatric disorders. T2—6 psychotic disorder (3 risperidone, 2 quetiapine, 1 olanzapine); 10 received mood stabilisers (4 valproate, 3 oxcarbamazepine, 3 gabapentin, 1 lithium)	1.5T	Brain Image	Total grey matter, total white matter, total brain, frontal lobe, temporal lobe, parietal lobe, cerebellum, amygdala, caudate	29	29	72.8	14.9	115.3	12.7	12.3	4.0	12.7	4.0	20	9	20	9
Jalbrzowski et al. 2017 [10]	Molecularly confirmed method—not specified	22q11.2DS group: 29 ASD and 4 psychotic disorder	3T	FreeSurfer	Amygdala	29	27	76.7	11.8	110.2	20.4	14.3	5.7	12.9	4.9	18	11	14	13
						32	28	81.5	14.0	110.2	20.4	13.8	5.4	12.9	4.9	14	18	15	13
Kates et al. 2001 [40]	FISH^a,b^	No diagnostic information available	1.5T	Automated Talairach atlas parcellation	Total grey matter, total white matter	10	10	73.0	15.0	96.6	10.4	10.1	1.8	10.1	1.9	3	7	3	7
Kates et al. 2005 [23]	FISH^a,b^	No diagnostic information available	1.5T	Measure	Frontal lobe	8	8	72.4	10.7	97.6	13.0	11.8	2.1	12.0	2.1	8	0	8	0
						11	10	72.4	10.7	97.6	13.0	11.8	2.1	12.0	2.1	0	11	0	10
Kates et al. 2011 [52]	FISH^a,b^	SIPS used but no diagnostic information available	1.5T	Brain Image	Total grey matter, total white matter, cerebellum, hippocampus, amygdala, lateral ventricles	36	24	70.9	13.9	94.5	14.0	11.8	2.1	11.8	1.9	19	17	15	9
						36	26	70.9	13.9	104.9	14.6	11.8	2.1	12.2	2.1	18	18	14	12
Lin et al. 2017 [36]	Molecularly confirmed method^c^	22q11.2DS group: 29 ASD; 4 Psychotic disorder and 27 ADHD	3T	FreeSurfer	Total grey matter, total white matter, hippocampus, caudate, lateral ventricles	66	56	78.7	12.5	111.5	19.0	15.7	7.6	14.6	6.9	32	34	31	25
Sandini et al. 2017 [84]	FISH^a,c^	22q11.2DS group: 7 psychosis; 21 ADHD; 15 any mood disorder; 42 anxiety disorders; 18 other psychiatric disorders and 15 psychotic disorder. Of these, 17 were on ≥1 psychotropic medication (10 methylphenidate, 10 anxiolytics, 12 antidepressants, 14 neuroleptics and 3 antiepileptics)	3T	FreeSurfer	Total grey matter, total white matter	108	96	74.5	12.7	109.8	12.6	18.5	8.6	18.0	5.0	49	59	49	47
Scott et al. 2016 [58]	FISH^a,c^	No diagnostic information available	3T	ANTS pipeline/ LoCA	Hippocampus	37	24	74.9	13.1	113.8	13.3	11.3	2.6	10.7	2.4	37	n/a	24	n/a
						32	24	74.9	13.1	113.8	13.3	11.3	2.6	10.7	2.4	n/a	32	n/a	24
van Amelsvoort et al. 2004 [85]	FISH^a,b^	22q11.2DS group: 13 schizophrenia (all with duration of illness >1 year and receiving antipsychotic medication. Further, 2 were hospitalised at the time of scanning)	1.5T	Measure	Total grey matter, total white matter, total brain, frontal lobe, temporal lobe, cerebellum, hippocampus, CFS, amygdala, caudate, lateral ventricles	13	6	69.0	8.0	75.0	16.0	34.0	11.0	36.0	10.0	6	7	4	3
						12	6	74.0	9.0	75.0	16.0	31.0	10.0	36.0	10.0	4	8	4	3

Where the study reports and analyses data separately for sub‐groups (such as males/females OR separate control/22q11DS groups) these are shown for each sub‐group

^a^Fluorescence in situ hybridisation

^b^No additional information provided

^c^Not specified

### Mean differences

We found significant overall decreases in mean volume in 22q11.2DS compared with controls for: total brain (*g* = −0.96; 95% CI, −1.26 to −0.65, *p* < 0.001); total grey matter (*g* = −0.81; 95% CI, −0.97 to −0.65, *p* < 0.001); and total white matter (*g* = −0.81; 95% CI, −0.98 to −0.64, *p* < 0.001), but no mean volume differences in cerebral spinal fluid (*g* = 0.10; 95% CI, −0.21 to 0.42, *p* = 0.519).

**Fig. 1:**
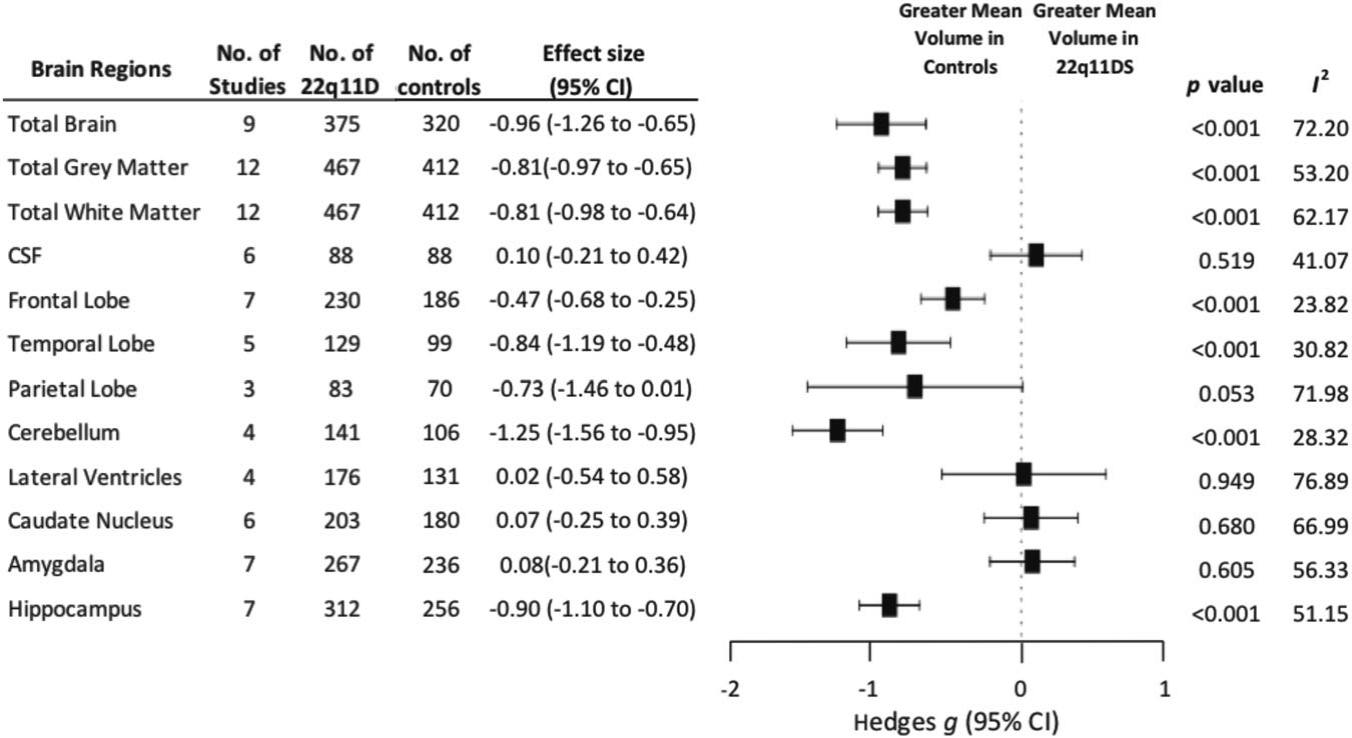
Mean volumes of total brain, total grey matter, total white matter, frontal lobe, temporal lobe, cerebellum and hippocampus were significantly reduced in individuals with 22q11.2DS. There were no significant mean differences between groups for cerebral spinal fluid (CSF), parietal lobe, lateral ventricles, caudate nucleus or amygdala. CL Confidence Interval; *P* indicates statistical significance; *I*^2^ indicates inconsistency.

When examining brain regions, there was an overall significant effect of group on mean volume (*x*^2^ = 148.25, *p* < 0.001). We also found significant overall decreases of mean volume in 22q11.2DS compared with controls in frontal lobe (*g* = −0.47; 95% CI, −0.68 to −0.25, *p* < 0.001), temporal lobe (*g* = −0.84; 95% CI, −1.19 to −0.48, *p* < 0.001), parietal lobe (*g* = −0.73; 95% CI, −1.46 to 0.01, *p* = 0.053), cerebellum (*g* = −1.25; 95% CI, −1.56 to −0.95, *p* < 0.001) and hippocampus (*g* = −0.90; 95% CI, −1.10 to −0.70, *p* < 0.001). There were no significant mean effects of group for lateral ventricles (*g* = 0.02; 95% CI, −0.54 to 0.58, *p* = 0.949), caudate nucleus (*g* = 0.07; 95% CI, −0.25 to 0.39, *p* = 0.680) or amygdala (*g* = 0.08; 95% CI, −0.21 to 0.36, *p* = 0.605) (see Fig. 1 and Supplementary Table 2). Pairwise interregional comparisons adjusted for multiple comparisons were also calculated (see Supplementary Table 3 for further details).

### Variability ratio

**Fig. 2:**
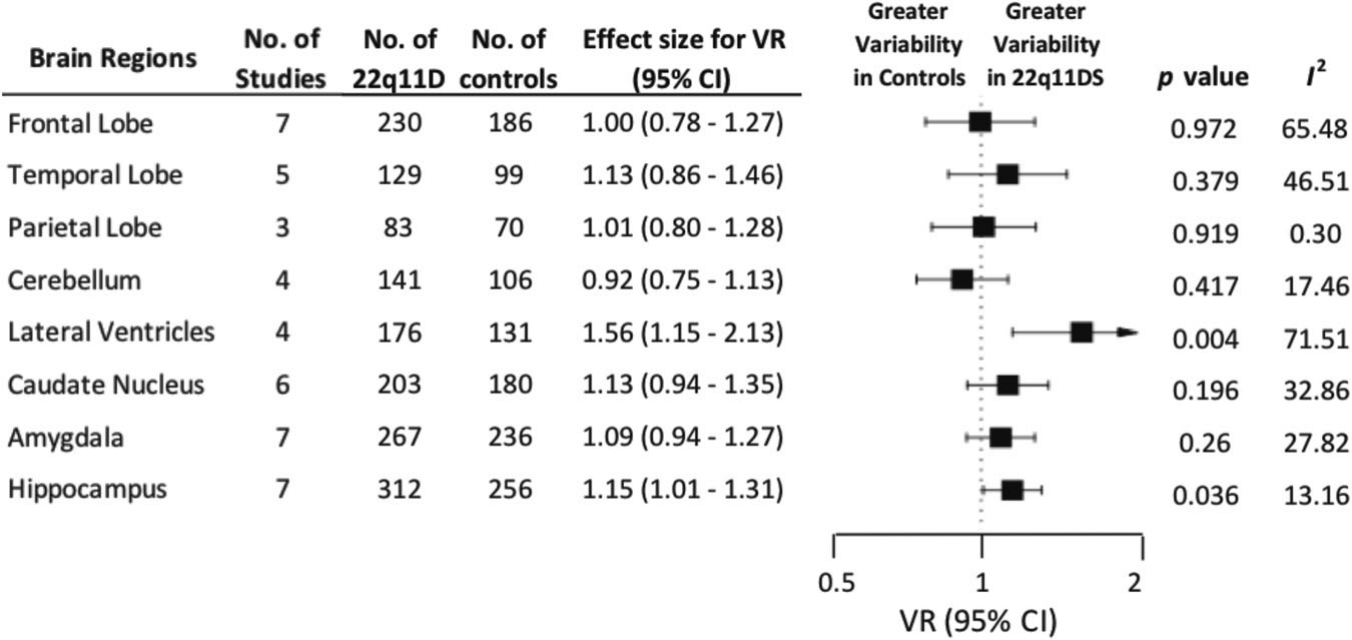
The variability ratio (VR) was significantly increased in the lateral ventricles and hippocampus, indicating greater variability in their volumes for individuals with 22q11.2DS. There were no significant VR for any other sub-regions. VR Variability Ratio; CL Confidence Interval; *P* indicates statistical significance; *I*^2^ indicates inconsistency.

In the brain regions we examined, we found significantly increased variability in 22q11.2DS individuals compared with controls for the hippocampus (VR, 1.15; 95% CI, 1.01–1.31; *p* = 0.036) and lateral ventricles (VR, 1.56; 95% CI, 1.15–2.13; *p* = 0.004). Variability was not significantly different between groups for any other regions: frontal lobe (VR, 1.00; 95% CI, 0.78–1.27; *p* = 0.972), temporal lobe (VR, 1.13; 95% CI, 0.86–1.46; *p* = 0.379), parietal lobe (VR, 1.01; 95% CI, 0.80–1.28; *p* = 0.919), cerebellum (VR, 0.92; 95% CI, 0.75–1.13; *p* = 0.417), caudate nucleus (VR, 1.13; 95% CI, 0.94–1.35; *p* = 0.196) or amygdala (VR, 1.09; 95% CI, 0.94–1.27; *p* = 0.260) (see Fig. 2 and Supplementary Table 2).

### Coefficient of variation ratio

**Fig. 3:**
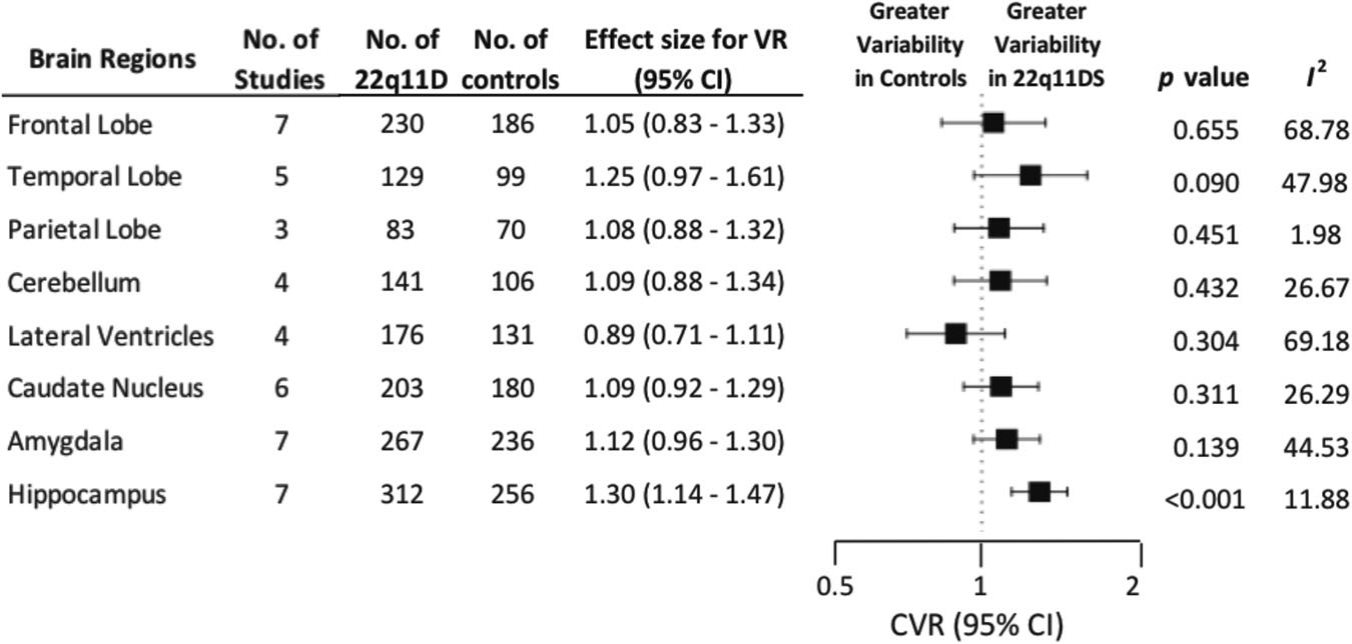
The coefficient of variability ratio (CVR) was significantly increased in hippocampus, indicating greater variability in volume for individuals with 22q11.2DS. There were no significant CVR for any other sub-regions. CVR Coefficient Variability Ratio; CL Confidence Interval; *P* indicates statistical significance; *I*^2^ indicates inconsistency.

There was increased variability in 22q11.2DS compared with controls remained significant when using CVR for the hippocampus (CVR, 1.30; 95% CI, 1.14–1.47; *p* < 0.001). However, this was not the case for the lateral ventricles (CVR, 0.89; 95% CI, 0.71–1.11; *p* = 0.304). Nor did variability, using coefficient ratio, become significant for any of the remaining regions; frontal lobe (CVR, 1.05; 95% CI, 0.83–1.33; *p* = 0. 655), temporal lobe (CVR, 1.25; 95% CI, 0. 97–1.61; *p* = 0.090), parietal lobe (CVR, 1.08; 95% CI, 0.88–1.32; *p* = 0.451), cerebellum (CVR, 1.09; 95% CI, 0.88–1.34; *p* = 0.432), caudate nucleus (CVR, 1.09; 95% CI, 0.92–1.29; *p* = 0.311) or amygdala (CVR, 1.12; 95% CI, 0.96–1.30; *p* = 0.139) or (see Fig. 3 and Supplementary Table 2).

### Meta-regression/sensitivity analysis

Age was not associated with regional mean volume differences (*p* > 0.05 for all regions). Interestingly, we found that sex was associated with the magnitude of mean volume differences in frontal lobe (*z* = −2.38, *p* = 0.017) and lateral ventricles (*z* = 3.12, *p* = 0.013), with studies containing a greater proportion of females showing greater reductions in brain volumes (see Supplementary Fig. 2). In addition, IQ was associated with lateral ventricles mean volume. Studies with greater differences in IQ between 22q11.2DS and healthy controls showed larger increase of lateral ventricle volume for the 22q11.2DS group (*z* = 3.12, *p* = 0.002) (see Supplementary Fig. 3).

However, there was no significant relationship between variability of age or IQ with hippocampal variability (*z* = 0.02, *p* = 0.86; *z* = 0.118, *p* = 0.578, respectively) (see Supplementary Fig. 4), nor with variability in lateral ventricles (*z* = 0.028, *p* = 0.87; *z* = −0.068, *p* = 0.845, respectively) (see Supplementary Fig. 5).

When we conducted the analysis, excluding individuals with 22q11.2DS with psychiatric comorbidities and/or psychotropic medication, results remained similar both for mean volume differences and variability (see Supplementary Results).

### Publication bias and inconsistency

Using a multivariate analogue of Egger’s test [33], we found that funnel plot asymmetry was significant for Hedges g (*x*^2^ = 25.98, *p* = 0.011), but not for lnVR (*x*^2^ = 0.48, *p* = 0.975), nor lnCVR (*x*^2^ = 2.107, *p* = 0.716) (see Supplementary Fig. 5).

Inconsistency (between-study heterogeneity), as measured by *l*^2^, depending on brain regions, ranged from 23.82 to 76.89 for Hedges g, 0.30 to 71.51 for lnVR and 1.98 to 69.18 for lnCVR (Figs. 1–3).

## Discussion

Our first finding was a widespread volumetric reduction in 22q11.2DS compared with typically developing controls in total brain, total grey and total white matter with large effect sizes. Further, we identified regionally specific decreases in the volume of frontal and temporal lobe, as well as in subcortical regions such as hippocampus. Secondly, we identified the hippocampus as a region of uniquely increased variability in 22q11.2DS.

### Brain volume reductions in 22q11.2 deletion

Reductions in grey and white matter volume in 22q11.2DS compared with controls have been a consistent finding across studies. More recently, grey matter differences have been examined as its two independent components, cortical thickness and surface area [19, 22, 25, 36], with a study by the ENIGMA 22q11.2DS consortium analysing the largest sample to date (*n* = 474) [17], and finding reduced cortical volume in 22q11.2DS, primarily driven by reduced surface area, whereas increases were found in cortical thickness [17]. Similarly, studies have observed white matter reductions in this population, which might indicate potential impairments in myelination, which could contribute to abnormalities in brain circuitry. For example, a recent large multicentre diffusion tensor imaging study from the ENIGMA 22q11.2DS working group found decreased diffusivities, with smaller and undulated axons in 22q11.2DS compared with healthy controls [37]. As such, the aberrant axonal differentiation and migration in 22q11.2DS may lead to the reduction in gyral complexity in the syndrome, which could drive the regional decreases in surface area [37]. Further, in the 22q11.2 locus, there are genes related to axonal migration, shaping brain morphology (i.e. *PIK4CA* and *RTN4R*) [38, 39] and when the microdeletion occurs, this may contribute to the observed white matter abnormalities [24, 40].

Our results extend the findings of the original meta-analysis [15], by both including a larger sample, and studies with more updated methodological approaches. In the present study, we found reduction in cortical and subcortical regions including frontal, temporal, parietal lobe, cerebellum and hippocampus. When we conducted the omnibus test and pairwise comparisons, the effect size in the volume of frontal lobe volume was smaller than that in the cerebellum. No significant differences were observed between frontal and temporal lobe or temporal lobe and cerebellum. This outcome is only partly in agreement with the rostro-caudal gradient (frontal < temporal < cerebellum) that has been shown in previous studies, which may be explained by increased sample size, or differences in methodological approaches e.g. use of multivariate vs. univariate analysis. Nonetheless, the finding of frontal < cerebellum is consistent with an aberrant developmental trajectory along the anterior–posterior axis early in developmental years [15, 16], potentially secondary to disruption of genes that encode neurodevelopmental morphogens that are implicated in the rostro-caudal axis [41].

Given the increased rates of neuropsychiatric disorders in 22q11.2DS, drawing parallels with studies examining individuals at high clinical and genetic risk for developing psychosis and/or ASD is crucial to enhance understand the neurodevelopmental phenotype of this microdeletion. For example, a meta-analysis in individuals at high risk of developing psychosis found structural abnormalities in similar regions to those found in this study, with reduced volume of middle and superior temporal gyri, middle frontal gyrus, hippocampus, parahippocampus and anterior cingulate cortex [42]. Comparably, studies at genetic high risk groups for psychosis (including unaffected first degree relatives and twins discordant for schizophrenia) have shown volumetric decreases in frontal lobe [43–45], temporal lobe [46], hippocampus [43, 44, 47], parahippocampus [43, 48, 49], as well as increases of lateral and third ventricles [43, 47]. Further metanalytic evidence in first episode psychosis has shown an inverse correlation between grey matter volume in temporal lobe and severity of psychotic symptoms [48]. It is worth noting that there was a larger magnitude of the effect sizes of mean differences in our study compared with the ones previously reported in genetic high-risk groups for psychosis [50] and in schizophrenia [26]. In addition, the most recent meta-analysis by the ENIGMA Schizophrenia working group showed widespread cortical thinning with regional specificity and decrease in surface area without specificity in psychosis [51]. Frontal and temporal lobe were the brain regions with the largest effect sizes for both measures, with specificity only for cortical thickness, but not for surface area [51].

Similarly, in 22q11.2DS, a longitudinal study following up adolescents over a 3-year period found an inverse correlation between volume in the prefrontal, temporal lobe and cerebellum with severity of total prodromal psychotic symptoms [52]. In this study, only decrease of grey matter volume in temporal lobe was associated with increase of positive prodromal psychotic symptoms [52]. Moreover, when comparing individuals with 22q11.2DS with or without schizophrenia, Chow et al. showed an association of the disorder with reduction of superior temporal gyrus [18]. Further, a recent large-scale study in 22q11.2DS found increased cortical thickness, along with decreased surface area in 22q11.2DS relative to control [17], which is in contrast with the widespread cortical thinning that has been found in idiopathic schizophrenia [51]. However, there was significant convergence of affected brain regions between 22q11.2DS and idiopathic schizophrenia. Individuals with 22q11.2DS and psychosis showing cortical thinning in fronto–temporal regions compared with the ones without psychosis, with effect sizes similar to the ones observed in idiopathic schizophrenia [17]. Combined, these findings might suggest that in individuals with 22q11.2DS, frontal and medial temporal lobe grey matter loss linked to cortical thinning may serve as a vulnerability marker for psychosis [53].

Likewise, in the ASD literature, several of the observed regions in this study have been highlighted. For example, there is evidence that frontal and temporal regions appear to be more affected than parietal and occipital regions, suggesting that the temporal sequence of typical early brain development (i.e. back to front) is perturbed in ASD [54]. A recent large-scale study from the ENIGMA-ASD working group has shown decreased volume in striatum, amygdala and hippocampus, enlarged lateral ventricles, increased frontal thickness and decreased temporal thickness with small to moderate effect sizes in ASDs relative to controls [55]. Our results partially overlap with these findings, for example, by showing reductions in hippocampus. Although there have been few studies in 22q11.2DS individuals with ASD, the findings to date have implicated volumetric difference in the amygdala [10, 12], decreased cortical thickness in bilateral parahippocampus [10] and increased cortical volume and surface area in right parieto–temporal regions, and left posterior cingulate and dorsolateral prefrontal cortex [25]. However, in view of the limited number of structural imaging studies investigating ASD on 22q11.2DS, any interpretations about convergence of affected regions between these disorders should be made cautiously.

In summary, decrease in grey matter volume in frontal and medial temporal lobe associated with cortical thinning in 22q11.2DS may suggest increased vulnerability to psychosis. It still remains unclear whether structural abnormalities in 22q11.2DS may help us explain the increased risk for ASD. This becomes more complicated given that idiopathic ASD and schizophrenia share some neuroanatomical abnormalities, as well as the high rates of comorbidity of these disorders in the 22q11.2DS population. Future longitudinal studies in 22q11.2DS are needed to disentangle the degree that these disorders share neuroanatomical variation, and to establish common or distinct genetic or molecular mechanisms across disorders.

### Variability

In the second part of our study, we demonstrated greater volumetric variability in the group of 22q11.2DS compared with the group of typically developing controls in hippocampus and lateral ventricles; with this remaining significant for the hippocampus when controlling for mean volume of the region.

There are several potential explanations for this finding. For example, it is possible that variability differences may reflect heterogeneous biological mechanisms in 22q11.2DS, suggesting that brain regions are affected differently across carriers. In support of this notion, a large-scale study on 22q11.2DS has found that deletion size impacts on brain structure, in particular for cortical surface area [17]. The increased variability in hippocampus and lateral ventricles may imply that these brain areas are affected only in some patients or to a varying extent across carriers. The hippocampus plays a key role in memory and cognition and aberrant morphology has been shown in schizophrenia, ASD and 22q11.2DS. Previous studies in mice models of 22q11.2DS have shown reduced neurogenesis [56] and density of dendritic spines in hippocampus [57], suggesting that these processes may lead to morphological alterations observed in 22q11.2DS. Moreover, it has been suggested, that in children with 22q11.2DS, the reduction in hippocampal volume might be due to greater variation in the shape of the anterior hippocampus, and a greater inward deformation [58]. In addition, it has been demonstrated that hippocampus, particularly ventral hippocampus, can regulate dopamine function via involvement of glutamatergic input to nucleus accumbens that leads to increase of GABAergic activity to the ventral pallidum [59]. Furthermore, evidence from preclinical and clinical data has shown that hyperactivity of hippocampus in schizophrenia can lead to increased tonic dopamine firing and hyperdopaminergia [60, 61]. More recently, a longitudinal study in 22q11.2DS showed a volumetric decrease of hippocampus, with individuals with 22q11.2DS and psychotic symptoms presenting with a further reduction of volume during adolescence, a crucial period for the emergence of psychosis [62]. All subfields in hippocampus were similarly affected, except CA2/3, which was interestingly decreased in 22q11.2DS and psychosis, suggesting that this atrophy is associated with the appearance of psychotic symptoms [62]. In view of the above, it can be speculated that hippocampal abnormalities in 22q11.2DS may be more common in those who develop psychosis. Thus, variability in the neurobiology affecting the hippocampus may contribute to the clinical heterogeneity, and explain why not all individuals with 22q11.2DS develop psychosis, consistent with differing neurodevelopmental trajectories underlying psychosis [63]. On the other hand, there seems to be less evidence to support a link between variability in hippocampus and ASD.

Notably, we observed unaltered variability in frontal, temporal, parietal, amygdala, cerebellum and caudate regions, which suggest that these are affected consistently among patients. Brugger et al. reported greater volumetric variability in individuals with first episode psychosis in putamen, temporal lobe, thalamus and third ventricle, unaltered variability in frontal lobe and caudate, and reduced variability in anterior cingulate cortex, supporting neurobiological heterogeneity in this disorder and highlighting the anterior cingulate cortex as a core component of the biological processes across schizophrenia subtypes [26]. Our results suggest a level of convergence with the previous variability meta-analysis, showing unaltered variability of frontal lobe and caudate and increased variability in hippocampus. However, when comparing the studies, it is important to bear in mind, that individuals included in our meta-analysis, were much younger and therefore, less likely to have developed psychotic symptoms/psychosis. Therefore, any interpretation should be made cautiously.

Another possible explanation is that the variability differences we observe may be due to homogeneity of the control groups, who are often unusually healthy, as the presence of significant illness are often recruitment exclusion criteria for controls, while individuals with 22q11.2DS are at increased risk of numerous physical and mental comorbidities [64]. This is, of course, an issue in general to case–control research across medicine, however the concern is more acute in relation to measures of variability (as opposed to mean differences), as here, “the noise is the signal”. It is worth noting that in the majority of the studies we included in our analysis, IQ was higher in controls than in individuals with 22q11.2DS. In the meta-regression we conducted, we found that IQ had a significant effect on mean volume differences in lateral ventricles, however, there was no association between variability in hippocampus and/or lateral ventricles with variability in IQ. To our knowledge, no study has previously examined the effect of IQ on lateral ventricles volume in 22q11.2DS. Deboer et al. previously investigated the relationship between hippocampal volume and IQ in 22q11.2DS [65]. Authors showed that there was a strong association between hippocampal volume and Verbal IQ, which is relatively preserved in this group. In contrast, no correlation was found between hippocampal volume with Performance IQ [65]. Thus, the lack of association between hippocampal variability with variability in IQ in our study does not exclude the possibility of relationship between hippocampal variability with specific cognitive functions. However, we were unable to test this due to the small number of studies providing this information. Moreover, we found no association between age variability and variability in either hippocampus or lateral ventricles, which may not be surprising given that groups in the studies were matched for age.

### Limitations and future directions

First, there was evidence for moderate-to-high inconsistency of effect size estimates for mean volume differences for some brain regions, with parietal lobe having the highest inconsistency (i.e. *I*^2^ = 72%). However, there were only three studies for this region, thus a relatively large effect size confidence interval for this measure. Although the meta-regression indicated that age did not account for the observed inconsistency, sex did significantly influence our findings for mean volumetric differences in frontal lobe and lateral ventricles. Studies with a greater proportion of females had greater reduction in brain volume. In addition, IQ was associated with the mean volumetric differences for lateral ventricles, with studies with a greater difference in IQ between the two groups showing larger lateral ventricle volumes in the patient group. For the variability analysis, inconsistency was found in frontal lobe and lateral ventricles (i.e. *I*^2^ = 74%, both for VR and CVR). However, the random effects model was applied in our analysis, which takes into account inconsistency.

In the sensitivity analysis, we demonstrated that diagnosis did not influence our results for either mean volume differences or variability.

Second, our regression test showed asymmetry of the funnel plot for mean volumetric differences, which may represent a degree of publication bias. In contrast, we did not find significant asymmetry for lnVR or lnCVR, which is perhaps unsurprising as variability is rarely a primary outcome in imaging studies.

Third, as the majority of the studies in our analysis included young individuals and adolescents, it is not clear whether we can extrapolate our results to the adult population of 22q11.2DS. It is important to consider different neurodevelopmental stages when comparing individuals with 22q11.2DS, as rates of cortical development are not linear across lifespan, and it is unclear at which exact stages it might be disrupted in this population. Moreover, due to insufficient number of studies to conduct a meta-analysis in some brain regions that have been involved previously in 22q11.2DS, such as occipital lobe [66, 67], anterior cingulate cortex [19, 68], insula [69], it would be crucial in the future to expand this analysis in these regions when more data are available, as well as in current regions (i.e. parietal), where only a small number of studies was available.

Last, many of the studies included in this meta-analysis examined large regions of interest and used techniques, which do not compare regions on a voxel-wise basis. In contrast, recent studies deploy the Freesurfer software (http://surfer.nmr.mgh.harvard.edu/) which enables accurate and robust registration and parcellation of cortical regions [70]. Similarly, a vertex-based approach has recently being employed in the analysis in an attempt to detect subtle and spatially distributed differences across regions [25].

Currently, large-scale multicentre imaging studies are being conducted, as part of the ENIGMA 22q11.2DS consortium, using advanced approaches (e.g. FreeSurfer) for the analysis of grey and white matter structures in this microdeletion. Results to date have shown the size of the 22q11.2 deletion can influence brain structure [17]. In one of these studies, reduction in cortical grey matter volume was found, driven by reductions in surface area, whereas cortical thickness was increased [17]. Further, the spatial pattern of thicker cortex resembled that of surface area reduction [17]. Similarly, there were large effects of the 22q11.2 deletion on white matter microstructure, such as widespread reductions in mean, axial and radial diffusivities in 22q11.2DS, especially in regions with major cortico-cortical and cortico-thalamic fibres [37]. Authors proposed that the pattern of abnormalities observed may reflect disrupted neurogenesis of projection neurons in outer cortical layers [37]. However, none of these studies have yet investigated the neuroanatomical variability within regions. Future studies should, where possible, examine this in more detail, both in regards to grey and white matter, as well as across regions in an attempt to detect more subtle and spatially distributed differences.

## Conclusions

In summary, we found that 22q11.2DS is associated with both global and regional volumetric decrease in total brain, total grey and white matter, frontal, temporal, parietal lobe, cerebellum and hippocampus. Our meta-analysis further suggests that there might be a convergence in neuroanatomical abnormalities between 22q11.2DS and what has previously been found in schizophrenia and to some degree in ASD. Finally, the increased variability in hippocampus in 22q11.2DS may explain some of the neuropsychiatric heterogeneity we observed in this genetic mutation and may be more likely associated with the emergence of psychosis. Future large-scale structural imaging studies are required to test the potential utility of the increased variability of hippocampus for stratification or prognostic biomarker in 22q11.2DS.

## Supplementary information

Supplementary material
