# Supplementary material for: Magnitude and heterogeneity of brain structural abnormalities in 22q11.2 deletion syndrome: a meta-analysis
Source: Mol Psychiatry. 2020 Jan 10;25(8):1704–17. doi: 10.1038/s41380-019-0638-3 (PMC7387301; doi:10.1038/s41380-019-0638-3)

**Supplementary information**

**Contents:**

**Page 2-3:** Supplementary results

**Page 4:** Supplementary Figure 1: Flow chart of the inclusion of studies for the meta-analysis

**Page 5:** Supplementary Table 1: Correlation coefficient for each combined left and right hemisphere brain region.

**Pages 6-9:** Supplementary Table 2: Effect sizes & Variability for each individual study and subgroup per region

**Page 10:** Supplementary Table 3: Pair-wise interregional comparisons

**Pages 11:** Supplementary Figure 2: Meta regression for mean brain volume differences with sex

**Pages 12:** Supplementary Figure 3: Meta regression for mean brain volume differences in lateral ventricles with IQ

**Page 13:** Supplementary Figure 3: Metaregression for Variability in Hippocampus with Age and IQ variability

**Page 14**: Supplementary Figure 4: Metaregression for Variability in Lateral Ventricles with Age and IQ variability

**Pages 15-16:** Supplementary Figure 5: Publication Bias

**Supplementary Results**

**Sensitivity analysis**

To examine whether diagnosis influced our results, we conducted sensitivity analysis excluding the participants with 22q11.2 deletion with psychiatric cormobidities and/or on psychotropic medication.

**Mean volume Differences**

Our results remained robust for overall mean volume decreases in 22q11.2DS compared to controls: total brain (*g* = -0.94; 95% CI, -1.27 to -0.6, *p* < 0.001); total grey matter (*g* = -0.81; 95% CI, -1.04 to -0.57, *p* < 0.001); and total white matter (*g* = -0.8; 95% CI, -1.07 to -0.53, *p* < 0.001), but no mean volume differences in cerebral spinal fluid (*g* = -0.096; 95% CI, -0.45 to 0.26, *p* = 0.6).

In agreement with our previous results, significant overall reduction of mean volume in 22q11.2DS compared to the control group was observe in frontal lobe (*g* = -0.48; 95% CI, -0.77 to -0.18, *p* =0.002), temporal lobe (*g* = -0.89; 95% CI, -1.27 to -0.51, *p* < 0.001), parietal lobe (*g* = -0.85; 95% CI, -1.54 to -0.15, *p* = 0.017), cerebellum (*g* = -1.21; 95% CI, -1.51 to -0.91, *p* < 0.001) and hippocampus (*g* = -0.91; 95% CI, -1.17 to -0.65, *p* < 0.001). There were no significant mean effects of group for lateral ventricles (*g* = -1.3; 95% CI, -2.6 to -0.024, *p =* 0.056), caudate nucleus (*g* = -0.11; 95% CI, -0.46 to 0.23, *p* = 0.52) or amygdala (*g* = 0.02; 95% CI, -0.27 to 0.3, *p* = 0.89).

**Variability Ratio**

In concordance with our previous findings, there was significantly increased variability in the hippocampus in 22q11.2DS individuals compared to controls (VR, 1.15; 95% CI, 1– 1.3; *p* = 0.047). However, variability for lateral ventricles did not remain statistically significant (VR, 0.9 95% CI, 0.28 – 2; *p* = 0.86).

Results for the other brain regions remained non significant: frontal lobe (VR,0.96; 95% CI, 0.73 – 1.3; *p* = 0.84), temporal lobe (VR, 0.92; 95% CI, 0.87 – 1.4; *p* = 0.6), parietal lobe (VR, 0.99; 95% CI, 0.79 – 1.24; *p* = 0.9), cerebellum (VR, 0.97; 95% CI, 0.76 – 1.19; *p* = 0.8), caudate nucleus (VR, 1.04; 95% CI, 0.77 – 1.3; *p* = 0.76) or amygdala (VR, 1.06; 95% CI, 0.92 – 1.2; *p* = 0.4).

**Coefficient of Variation Ratio**

In line with our findings, when using CVR, we found increased variability for the hippocampus in 22q11.2DS compared to controls (CVR, 1.29; 95% CI, 1.15 – 1.43; *p* < 0.001). Results remained non significant for all other brain regions; lateral ventricles (CVR, 0.75; 95% CI, 0.44 – 1.17; *p* = 0.24), frontal lobe (CVR, 1.04; 95% CI, 0.7 – 1.38; *p* = 0.82), temporal lobe (CVR, 1.2 95% CI, 0. 87 – 1.52; *p* = 0.23), parietal lobe (CVR, 1.08; 95% CI, 0.87 – 1.28; *p* = 0.48), cerebellum (CVR, 1.11; 95% CI, 0.9 – 1.33; *p* = 0.3), caudate nucleus (CVR, 1.08; 95% CI, 0.83 – 1.33; *p* = 0.54) or amygdala (CVR, 1; 95% CI, 0.96 – 1.23; *p* = 0.2).

**Supplementary Figure 1: Flow chart of the inclusion of studies for the meta-analysis**

#133 full-text articles excluded, with reasons:

- Overlapping samples
- Conference Abstracts
- Wrong units (i.e. cm3)
- Different methods
- No control groups
- Missing SD for means

#6911 records identified through database searching

#4386 duplicates removed

#2525 titles screened

#306 abstracts assessed for eligibility

#2219 records excluded

#157 full-text articles assessed for eligibility

**Identification**

**Screening**

**Included**

**Eligibility**

#24 studies included in quantitative synthesis (meta-analysis)

#149 records excluded

**Supplementary Table 1: Correlation coefficient for each combined left and right hemisphere brain region**

**Supplementary Table 2: Effect sizes & Variability for each individual study and subgroup per region**

Total Brain

| **Study** | **Mean Effect size** | | **Variability** | | | |
| --- | --- | --- | --- | --- | --- | --- |
|  | **Hedges g** | **Hedges g CI** | **VR** | **VR CI** | **CVR** | **CVR CI** |
| Antshel et al., 2008 | -0.42 | [-0.75, -0.09] | 0.99 | [0.78, 1.25] | 1.03 | [0.82, 1.29] |
| Bearden et al., 2007 | -2.59 | [-3.51, -1.66] | 0.63 | [0.38, 1.04] | 0.67 | [0.41, 1.12] |
| Campbell et al., 2006 | -0.64 | [-1.15, -0.13] | 0.90 | [0.63, 1.28] | 0.95 | [0.67, 1.36] |
| Debbane et al., 2006 | -1.17 | [-1.64, -0.70] | 1.10 | [0.81, 1.50] | 1.25 | [0.92, 1.69] |
| Dufour et al., 2008 | -0.99 | [-1.36, -0.61] | 1.35 | [1.05, 1.74] | 1.49 | [1.16, 1.92] |
| Eliez et al., 2001 | -1.44 | [-2.08, -0.79] | 1.10 | [0.72, 1.67] | 1.24 | [0.82, 1.88] |
| Glaser et al., 2007 | -0.92 | [-1.35, -0.50] | 1.08 | [0.81, 1.44] | 1.19 | [0.90, 1.59] |
| Gothelf et al., 2007 | -1.16 | [-1.72. -0.61] | 1.47 | [1.02, 2.13] | 1.66 | [1.15, 2.39] |
| van Amelsvoort et al., 2004 | -1.32 | [-2.37, -0.26] | 0.96 | [0.46, 2.00] | 1.10 | [0.53, 2.27] |
| van Amelsvoort et al., 2004 | -0.55 | [-1.54, 0.45] | 0.95 | [0.40, 1.08] | 0.90 | [0.43, 1.88] |

Total Grey Matter

| **Study** | **Mean Effect size** | | **Variability** | | | |
| --- | --- | --- | --- | --- | --- | --- |
|  | **Hedges g** | **Hedges g CI** | **VR** | **VR CI** | **CVR** | **CVR CI** |
| Baker et al., 2011 | -1.15 | [-2.14, -0.17] | 1.28 | [0.64, 2.56] | 1.45 | [0.75, 2.80] |
| Baker et al., 2011 | -1.38 | [-2.37, -0.38] | 1.30 | [0.66, 2.58] | 1.51 | [0.79, 2.88] |
| Bearden et al., 2009 | -1.35 | [-2.12, -0.59] | 1.04 | [0.63, 1.72] | 1.17 | [0.72, 1.90] |
| Chow et al., 2002 | -1.09 | [-1.92, -0.27] | 0.72 | [0.41, 1.27] | 0.83 | [0.49, 1.42] |
| Debbane et al., 2006 | -0.99 | [-1.44, -0.53] | 0.93 | [0.68, 1.26] | 1.05 | [0.78, 1.40] |
| Deboer et al., 2007 | -0.75 | [-1.22, -0.27] | 0.94 | [0.67, 1.31] | 1.01 | [0.74, 1.38] |
| Eliez et al., 2002 | -0.52 | [-1.03, 0.00] | 1.32 | [0.92, 1.90] | 1.40 | [0.99, 1.97] |
| Gothelf et al., 2007 | -0.80 | [-1.33, -0.26] | 1.71 | [1.18, 2.47] | 1.84 | [1.29, 2.62] |
| Kates et al., 2001 | -0.61 | [-1.51, 0.28] | 0.98 | [0.51, 1.89] | 1.05 | [0.57, 1.96] |
| Kates et al., 2011 | -0.35 | [-0.87, 0.17] | 0.82 | [0.57, 1.20] | 0.85 | [0.60, 1.22] |
| Kates et al., 2011 | -0.70 | [-1.22, -0.19] | 0.60 | [0.42, 0.87] | 0.65 | [0.46, 0.92] |
| Lin et al., 2017 | -0.58 | [-0.94, -0.22] | 1.09 | [0.84, 1.40] | 1.13 | [0.88, 1.44] |
| Sandini et al., 2017 | -1.02 | [-1.32, -0.73] | 0.99 | [0.81, 1.20] | 1.10 | [0.91, 1.33] |
| van Amelsvoort et al., 2004 | -0.06 | [-1.03, 0.91] | 1.40 | [0.67, 2.94] | 1.42 | [0.70, 2.85] |
| van Amelsvoort et al., 2004 | 0.09 | [-0.89, 1.07] | 1.34 | [0.64, 2.83] | 1.33 | [0.65, 2.69] |

Total White Matter

| **Study** | **Mean Effect size** | | **Variability** | | | |
| --- | --- | --- | --- | --- | --- | --- |
|  | **Hedges g** | **Hedges g CI** | **VR** | **VR CI** | **CVR** | **CVR CI** |
| Baker et al., 2011 | -1.27 | [-2.27, -0.27] | 0.70 | [0.35, 1.40] | 0.84 | [0.45, 1.57] |
| Baker et al., 2011 | -1.69 | [-2.73, -0.65] | 1.02 | [0.52, 2.03] | 1.22 | [0.65, 2.29] |
| Bearden et al., 2009 | -0.29 | [-0.99, 0.40] | 1.16 | [0.70, 1.93] | 1.22 | [0.78, 1.90] |
| Chow et al., 2002 | -0.24 | [-1.01, 0.53] | 0.68 | [0.39, 1.20] | 0.71 | [0.43, 1.17] |
| Debbane et al., 2006 | -0.74 | [-1.19, -0.30] | 1.21 | [0.89, 1.65] | 1.38 | [1.06, 1.80] |
| Deboer et al., 2007 | -0.65 | [-1.12, -0.17] | 1.10 | [0.79, 1.54] | 1.20 | [0.89, 1.62] |
| Eliez et al., 2001 | -1.64 | [-2.31, -0.97] | 0.81 | [0.54, 1.24] | 1.00 | [0.68, 1.45] |
| Gothelf et al., 2007 | -1.12 | [-1.67, -0.57] | 1.06 | [0.73, 1.54] | 1.25 | [0.90, 1.74] |
| Kates et al., 2001 | -0.74 | [-1.65, 0.16] | 1.31 | [0.68, 2.52] | 1.46 | [0.81, 2.61] |
| Kates et al., 2011 | -0.20 | [-0.72, 0.32] | 0.86 | [0.59, 1.24] | 0.88 | [0.63, 1.23] |
| Kates et al., 2011 | -0.57 | [-1.09, -0.06] | 0.86 | [0.60, 1.24] | 0.93 | [0.67, 1.29] |
| Lin et al., 2017 | -1.01 | [-1.39, -0.63] | 1.05 | [0.82, 1.36] | 1.12 | [0.88, 1.42] |
| Sandini et al., 2017 | -0.77 | [-1.06, -0.49] | 1.20 | [0.98, 1.46] | 1.32 | [1.11, 1.58] |
| van Amelsvoort et al., 2004 | -1.31 | [-2.36, -0.26] | 0.68 | [0.32, 1.42] | 0.81 | [0.42, 1.57] |
| van Amelsvoort et al., 2004 | -0.83 | [-1.85, 0.19] | 0.77 | [0.36, 1.62] | 0.87 | [0.45, 1.69] |

Cerebral Spinal Fluid

| **Study** | **Mean Effect size** | | **Variability** | | | |
| --- | --- | --- | --- | --- | --- | --- |
|  | **Hedges g** | **Hedges g CI** | **VR** | **VR CI** | **CVR** | **CVR CI** |
| Baker et al., 2011 | 0.54 | [-0.39, 1.48] | 1.59 | [0.80, 3.18] | 1.45 | [0.83, 2.52] |
| Baker et al., 2011 | -0.20 | [-1.11, 0.71] | 1.28 | [0.65, 2.53] | 1.33 | [0.77, 2.28] |
| Chow et al., 2002 | 0.68 | [-0.11, 1.47] | 0.95 | [0.54, 1.67] | 0.85 | [0.53, 1.34] |
| Deboer et al., 2007 | -0.23 | [-0.69, 0.24] | 1.35 | [0.97, 1.88] | 1.42 | [1.11, 1.82] |
| van Amelsvoort et al., 2004 | 0.66 | [-0.33, 1.65] | 2.11 | [1.01, 4.41] | 1.60 | [0.95, 2.70] |
| van Amelsvoort et al., 2004 | -0.08 | [-1.06, 0.90] | 0.95 | [0.45, 2.01] | 0.97 | [0.56, 1.70] |

Frontal lobe

| **Study** | **Mean Effect size** | | **Variability** | | | |
| --- | --- | --- | --- | --- | --- | --- |
|  | **Hedges g** | **Hedges g CI** | **VR** | **VR CI** | **CVR** | **CVR CI** |
| Baker et al., 2011 | 0.54 | [-0.39, 1.48] | 1.59 | [0.80, 3.18] | 1.45 | [0.83, 2.52] |
| Baker et al., 2011 | -0.20 | [-1.11, 0.71] | 1.28 | [0.65, 2.53] | 1.33 | [0.77, 2.28] |
| Chow et al., 2002 | 0.68 | [-0.11, 1.47] | 0.95 | [0.54, 1.67] | 0.85 | [0.53, 1.34] |
| Deboer et al., 2007 | -0.23 | [-0.69, 0.24] | 1.35 | [0.97, 1.88] | 1.42 | [1.11, 1.82] |
| van Amelsvoort et al., 2004 | 0.66 | [-0.33, 1.65] | 2.11 | [1.01, 4.41] | 1.60 | [0.95, 2.70] |
| van Amelsvoort et al., 2004 | -0.08 | [-1.06, 0.90] | 0.95 | [0.45, 2.01] | 0.97 | [0.56, 1.70] |

Parietal lobe

| **Study** | **Mean Effect size** | | **Variability** | | | |
| --- | --- | --- | --- | --- | --- | --- |
|  | **Hedges g** | **Hedges g CI** | **VR** | **VR CI** | **CVR** | **CVR CI** |
| Campbell et al., 2006 | 0.03 | [-0.46, 0.53] | 1.06 | [0.74, 1.51] | 1.05 | [0.78, 1.42] |
| Eliez et al., 2000 | -1.28 | [-2.07, -0.50] | 0.88 | [0.52, 1.49] | 1.02 | [0.64, 1.63] |
| Gothelf et al., 2007 | -1.22 | [-1.78, -0.66] | 1.02 | [0.70, 1.48] | 1.17 | [0.84, 1.63] |

Temporal lobe

| **Study** | **Mean Effect size** | | **Variability** | | | |
| --- | --- | --- | --- | --- | --- | --- |
|  | **Hedges g** | **Hedges g CI** | **VR** | **VR CI** | **CVR** | **CVR CI** |
| Bearden et al., 2004 | -0.32 | [-1.18, 0.53] | 1.26 | [0.67, 2.38] | 1.31 | [0.71, 2.41] |
| Campbell et al., 2006 | -0.52 | [-1.03, -0.02] | 0.81 | [0.56, 1.15] | 0.88 | [0.63, 1.23] |
| Eliez et al., 2001 | -1.25 | [-1.88, -0.62] | 1.22 | [0.80, 1.86] | 1.38 | [0.92, 2.07] |
| Gothelf et al., 2007 | -1.10 | [-1.65, -0.55] | 1.73 | [1.19, 2.50] | 1.95 | [1.37, 2.79] |
| van Amelsvoort et al., 2004 | -1.53 | [-2.61, -0.45] | 1.01 | [0.48, 2.12] | 1.23 | [0.60, 2.50] |
| van Amelsvoort et al., 2004 | -0.65 | [-1.65, 0.35] | 1.13 | [0.54, 2.40] | 1.23 | [0.60, 2.53] |

Cerebellum

| **Study** | **Mean Effect size** | | **Variability** | | | |
| --- | --- | --- | --- | --- | --- | --- |
|  | **Hedges g** | **Hedges g CI** | **VR** | **VR CI** | **CVR** | **CVR CI** |
| Eliez et al., 2000 | -1.13 | [-1.90, -0.36] | 0.96 | [0.57, 1.62] | 1.14 | [0.68, 1.89] |
| Gothelf et al., 2007 | -1.35 | [-1.92, -0.78] | 0.80 | [0.55, 1.15] | 0.93 | [0.65, 1.33] |
| Kates et al., 2011 | -0.90 | [-1.44, -0.36] | 1.06 | [0.73, 1.54] | 1.16 | [0.81, 1.67] |
| Kates et al., 2011 | -1.08 | [-1.62, -0.54] | 0.71 | [0.50, 1.03] | 0.81 | [0.57, 1.16] |
| van Amelsvoort et al., 2004 | -2.06 | [-3.23, -0.90] | 1.08 | [0.52, 2.26] | 1.39 | [0.67, 2.85] |
| van Amelsvoort et al., 2004 | -1.42 | [-2.50, -0.33] | 1.20 | [0.57, 2.54] | 1.43 | [0.69, 2.98] |

Caudate Nucleus

| **Study** | **Mean Effect size** | | **Variability** | | | |
| --- | --- | --- | --- | --- | --- | --- |
|  | **Hedges g** | **Hedges g CI** | **VR** | **VR CI** | **CVR** | **CVR CI** |
| Baker et al., 2011 | -0.53 | [-1.47, 0.40] | 1.05 | [0.53, 2.11] | 1.12 | [0.57, 2.22] |
| Baker et al., 2011 | -0.69 | [-1.62, 0.24] | 0.63 | [0.32, 1.26] | 0.71 | [0.36, 1.40] |
| Campbell et al., 2006 | 0.15 | [-0.34, 0.65] | 0.99 | [0.70, 1.42] | 0.97 | [0.68, 1.38] |
| Eliez et al., 2002 | 0.55 | [0.03, 1.06] | 1.11 | [0.77, 1.60] | 1.01 | [0.71, 1.45] |
| Gothelf et al., 2007 | -0.18 | [-0.69, 0.34] | 1.44 | [1.00, 2.09] | 1.48 | [1.03, 2.13] |
| Lin et al., 2017 | 0.72 | [0.35, 1.09] | 1.34 | [1.04, 1.73] | 1.25 | [0.97, 1.60] |
| van Amelsvoor et al., 2004 | -0.27 | [-1.24, 0.70] | 1.23 | [0.59, 2.58] | 1.30 | [0.63, 2.67] |
| van Amelsvoor et al., 2004 | -0.03 | [-1.01, 0.95] | 0.76 | [0.36, 1.61] | 0.77 | [0.37, 1.60] |

Hippocampus

| **Study** | **Mean Effect size** | | **Variability** | | | |
| --- | --- | --- | --- | --- | --- | --- |
|  | **Hedges g** | **Hedges g CI** | **VR** | **VR CI** | **CVR** | **CVR CI** |
| Baker et al., 2011 | -1.49 | [-2.51, -0.46] | 1.14 | [0.57, 2.28] | 1.38 | [0.70, 2.72] |
| Baker et al., 2011 | -2.43 | [-3.60, -1.26] | 1.31 | [0.66, 2.60] | 1.73 | [0.89, 3.37] |
| Debbane et al., 2006 | -1.12 | [-1.58, -0.66] | 1.04 | [0.77, 1.42] | 1.20 | [0.89, 1.63] |
| Eliez et al., 2001 | -0.85 | [-1.46, -0.25] | 1.29 | [0.85, 1.95] | 1.41 | [0.94, 2.13] |
| Kates et al., 2011 | -0.68 | [-1.21, -0.15] | 1.32 | [0.91, 1.92] | 1.44 | [1.00, 2.07] |
| Kates et al., 2011 | -0.68 | [-1.20, -0.16] | 1.33 | [0.92, 1.91] | 1.44 | [1.01, 2.06] |
| Lin et al., 2017 | -0.81 | [-1.18, -0.44] | 1.08 | [0.84, 1.39] | 1.17 | [0.91, 1.50] |
| Scott et al., 2016 | -0.82 | [-1.35, -0.28] | 1.00 | [0.69, 1.45] | 1.22 | [0.85, 1.75] |
| Scott et al., 2016 | -0.59 | [-1.13, -0.05] | 1.30 | [0.89, 1.90] | 1.49 | [1.02, 2.16] |
| van Amelsvoort et al., 2004 | -0.62 | [-1.61, 0.36] | 1.05 | [0.50, 2.19] | 1.17 | [0.57, 2.41] |
| van Amelsvoort et al., 2004 | -0.28 | [-1.26, 0.71] | 0.78 | [0.37, 1.65] | 0.82 | [0.39, 1.69] |

Amygdala

| **Study** | **Mean Effect size** | | **Variability** | | | |
| --- | --- | --- | --- | --- | --- | --- |
|  | **Hedges g** | **Hedges g CI** | **VR** | **VR CI** | **CVR** | **CVR CI** |
| Baker et al., 2011 | -0.42 | [-1.34, 0.51] | 1.36 | [0.68, 2.72] | 1.45 | [0.79, 2.67] |
| Baker et al., 2011 | -0.64 | [-1.57, 0.29] | 1.54 | [0.78, 3.04] | 1.68 | [0.92, 3.08] |
| Debbane et al., 2006 | -0.21 | [-0.64, 0.23] | 0.93 | [0.69, 1.27] | 0.97 | [0.75, 1.26] |
| Eliez et al., 2001 | -0.39 | [-0.98, 0.19] | 0.87 | [0.58, 1.33] | 0.94 | [0.66, 1.35] |
| Gothelf et al., 2007 | 0.14 | [-0.37, 0.66] | 1.00 | [0.69, 1.45] | 0.98 | [0.71, 1.35] |
| Jalbrzowski et al., 2017 | -0.70 | [-1.24, -0.16] | 0.94 | [0.65, 1.38] | 1.04 | [0.74, 1.45] |
| Jalbrzowski et al., 2017 | -0.65 | [-1.17, -0.13] | 1.03 | [0.72, 1.49] | 1.13 | [0.82, 1.57] |
| Kates et al., 2011 | 0.59 | [0.06, 1.11] | 0.88 | [0.61, 1.28] | 0.78 | [0.57, 1.08] |
| Kates et al., 2011 | 0.41 | [-0.10, 0.92] | 1.33 | [0.92, 1.91] | 1.24 | [0.90, 1.70] |
| van Amelsvoort et al., 2004 | -0.09 | [-1.06, 0.88] | 2.16 | [1.03, 4.51] | 2.20 | [1.13, 4.26] |
| van Amelsvoort et al., 2004 | 0.23 | [-0.76, 1.21] | 1.60 | [0.76, 3.38] | 1.54 | [0.78, 3.04] |

Lateral Ventricles

| **Study** | **Mean Effect size** | | **Variability** | | | |
| --- | --- | --- | --- | --- | --- | --- |
|  | **Hedges g** | **Hedges g CI** | **VR** | **VR CI** | **CVR** | **CVR CI** |
| Chow et al., 2002 | 1.47 | [0.61, 2.34] | 1.79 | [1.02, 3.15] | 0.95 | [0.64, 1.40] |
| Kates et al., 2011 | 0.68 | [0.15, 1.21] | 1.59 | [1.10, 2.31] | 0.72 | [0.48, 1.07] |
| Kates et al., 2011 | 0.77 | [0.25, 1.30] | 2.94 | [2.05, 4.23] | 1.26 | [0.96, 1.67] |
| Lin et al., 2017 | 1.05 | [0.67, 1.43] | 1.20 | [0.93, 1.55] | 0.67 | [0.57, 0.80] |
| van Amelsvoort et al., 2004 | 0.16 | [-0.81, 1.13] | 3.15 | [1.51, 6.59] | 2.46 | [1.22, 4.98] |
| van Amelsvoort et al., 2004 | -0.82 | [-1.83, 0.20] | 0.39 | [0.19, 0.83] | 0.58 | [0.35, 0.96] |

**Supplementary Table 3: Pair-wise interregional comparisons**

|  | **Amygdala**  ***p*-value** | **Caudate**  ***p*-value** | **Cerebellum**  ***p*-value** | **Frontal Lobe**  ***p*-value** | **Hippocampus**  ***p*-value** | **Lateral Ventricles**  ***p*-value** | **Parietal**  **Lobe**  ***p*-value** | **Temporal Lobe**  ***p*-value** |
| --- | --- | --- | --- | --- | --- | --- | --- | --- |
| **Amygdala** |  | 1 | <0.01 | 0.047 | <0.01 | 1 | 0.27 | <0.01 |
| **Caudate** |  |  | <0.01 | 0.09 | <0.01 | 1 | 0.23 | <0.01 |
| **Cerebellum** |  |  |  | <0.01 | 0.5 | <0.01 | 0.85 | 0.54 |
| **Frontal Lobe** |  |  |  |  | 0.08 | 0.76 | 0.99 | 0.63 |
| **Hippocampus** |  |  |  |  |  | <0.01 | 0.99 | 1 |
| **Lateral Ventricles** |  |  |  |  |  |  | 0.49 | 0.053 |
| **Parietal lobe** |  |  |  |  |  |  |  | 0.99 |

**Supplementary Figure 2: Meta regression for mean brain volume differences with sex**

1. **Frontal lobe effect size**

**
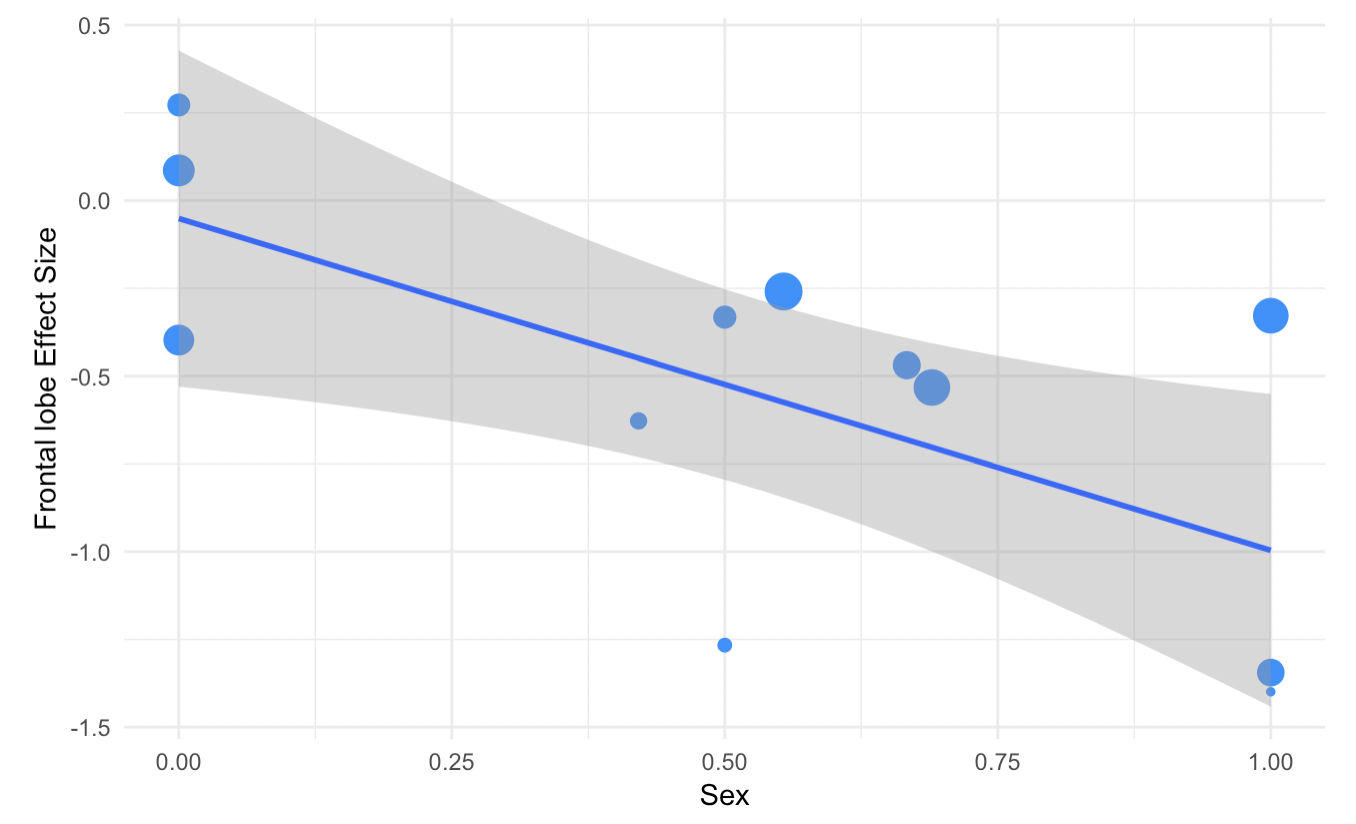
**

1. **Lateral ventricles effect size with sex**

**
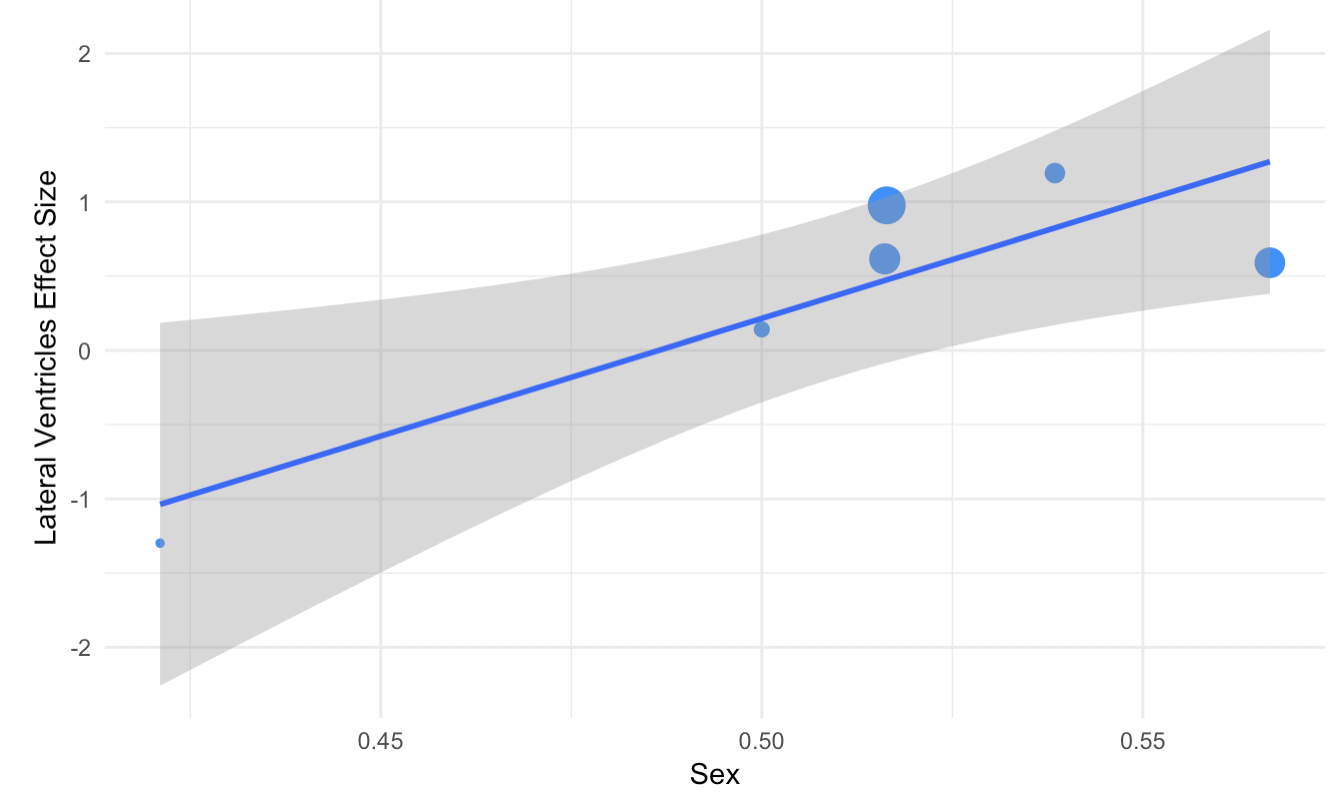
**

**Supplementary Figure 3: Meta regression for mean brain volume differences in lateral ventricles with IQ**

**
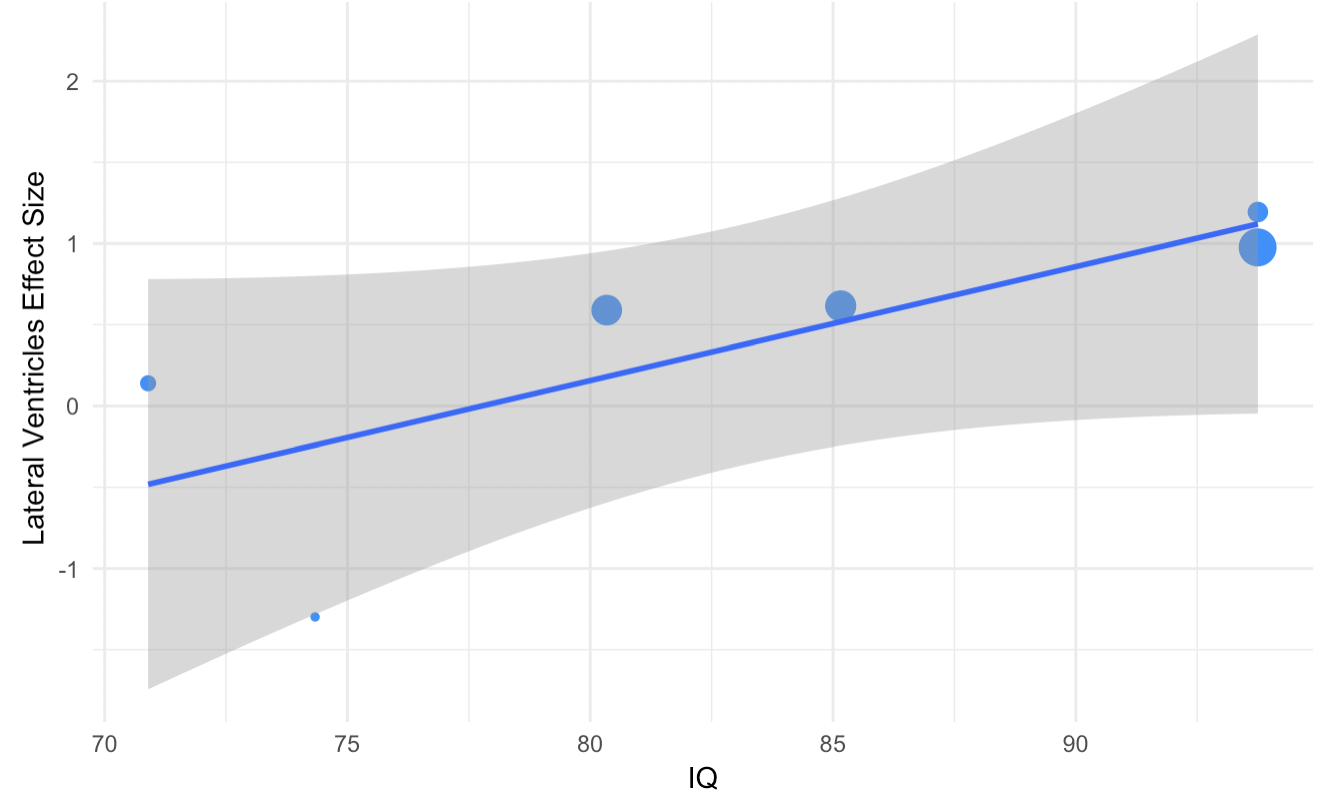
**

**Supplementary Figure 4 : Metaregression for Variability in Hippocampus with Age and IQ variability**

**Age**


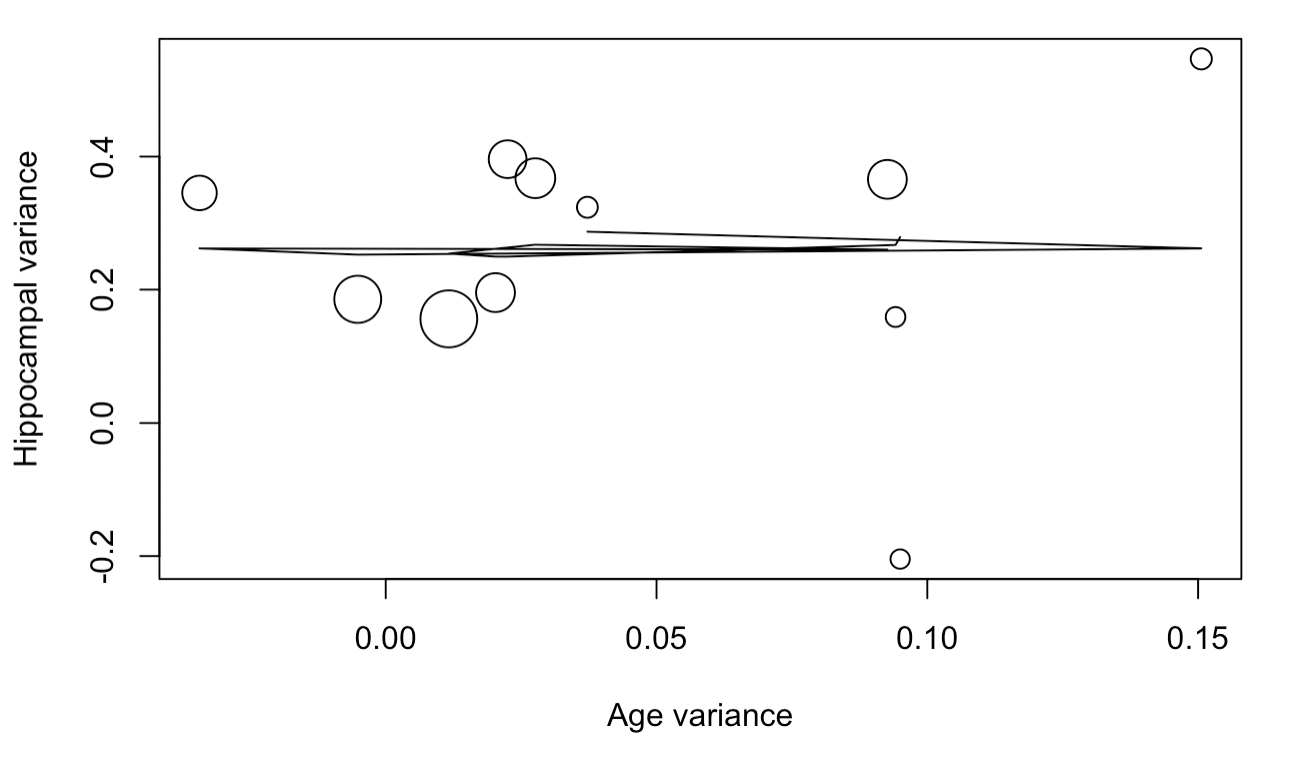


**IQ**

**
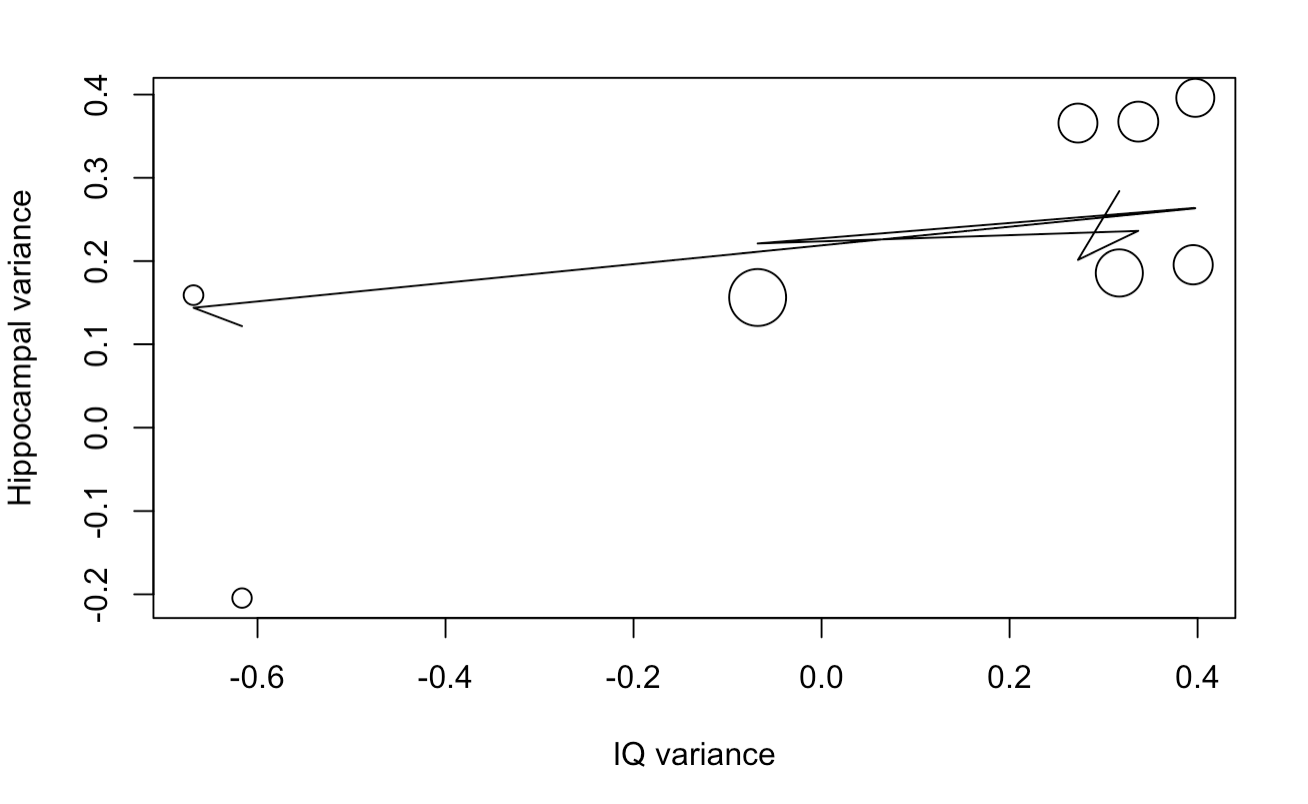
**

**Supplementary Figure 5: Metaregression for Variability in Lateral Ventricles with Age and IQ variability**

**Age**

**
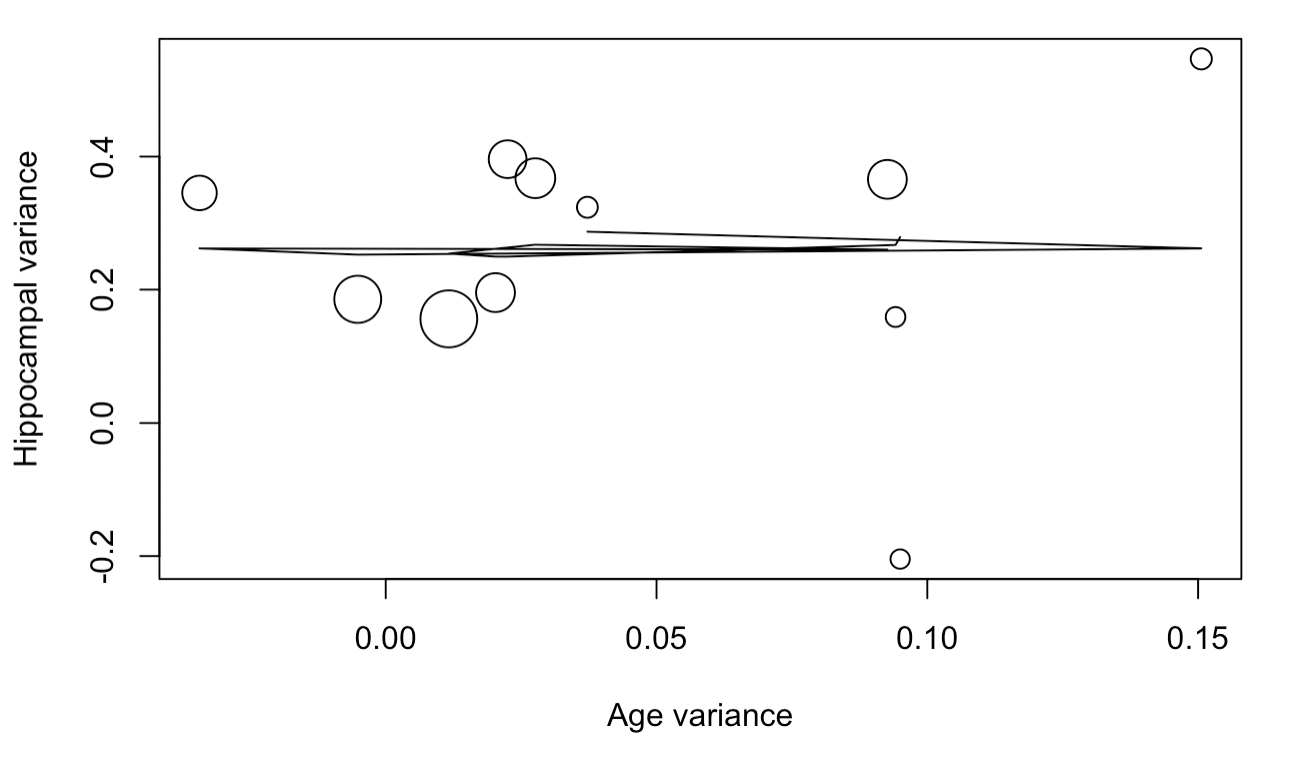
**

**
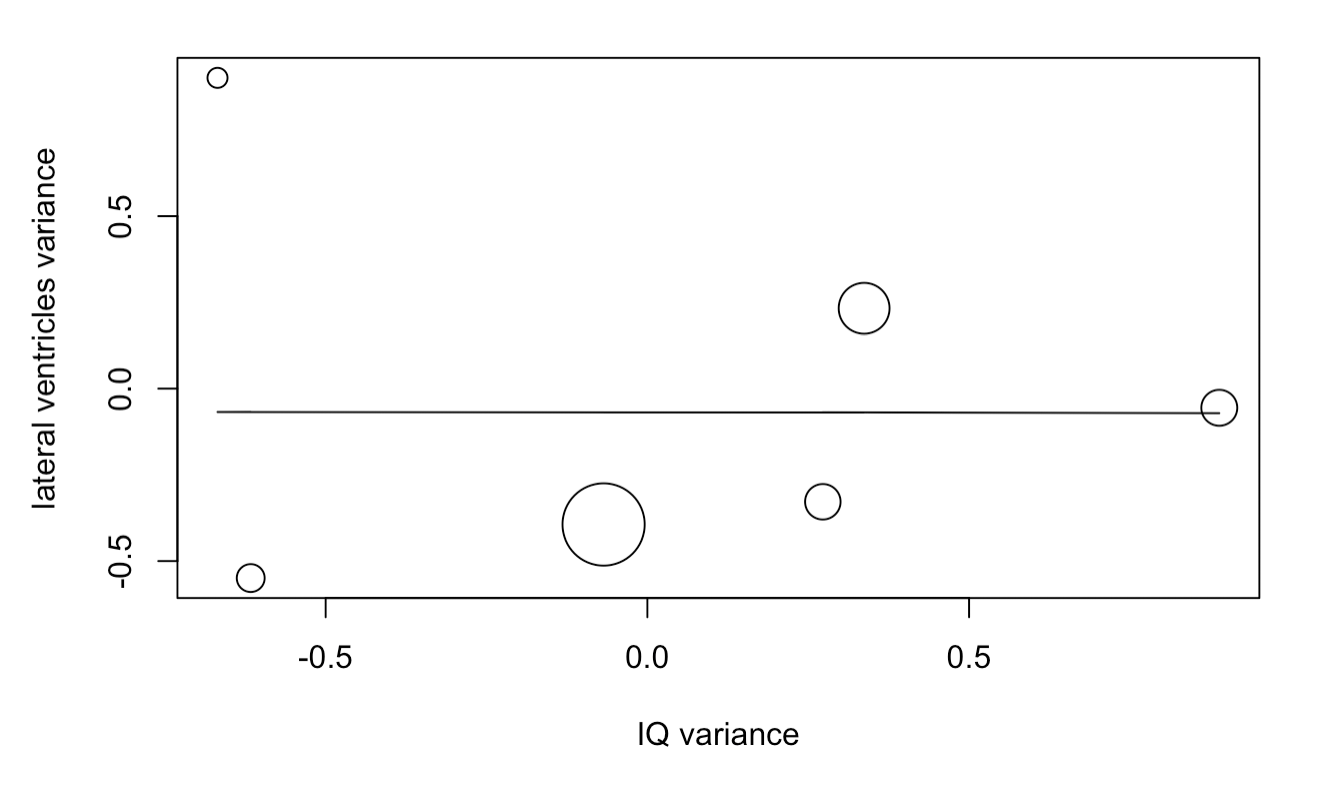
IQ**

**Supplementary Figure 5: Publication Bias**

Funnel plot for Means

**
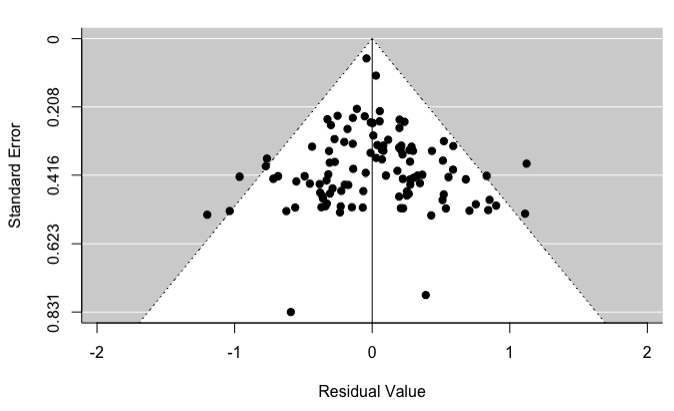
**

Funnel Plot for Variability Ratio


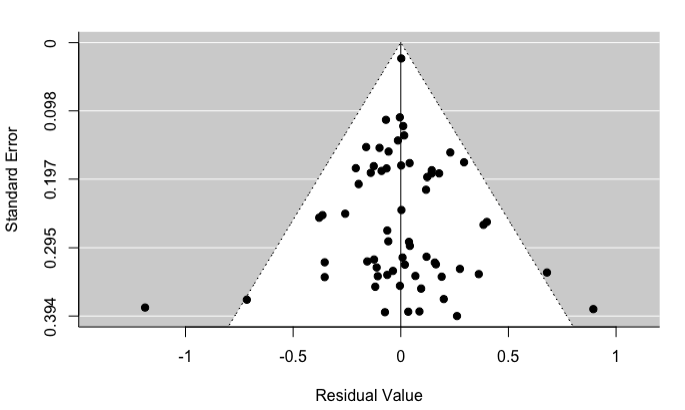


Funnel Plot for Coefficient Variability Ratio


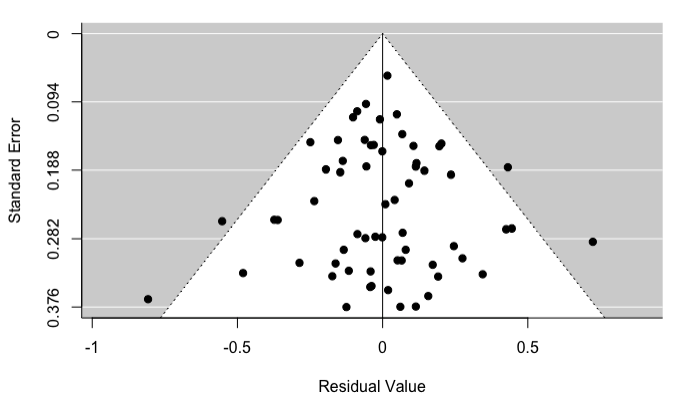

Supplement: Supplementary file 1 — Supplementary material [file 41380_2019_638_MOESM1_ESM.docx]
